# Leveraging Quantitative Proteomics and Extracellular Vesicle Data to Uncover Druggable Receptor Kinases Across Cancers

**DOI:** 10.1002/jev2.70275

**Published:** 2026-04-14

**Authors:** Jina Kim, Su Yeon Yeon, Kyerim Choi, Hojung Kim, Hyoyoung Kim, Daehee Hwang, Sungyong You

**Affiliations:** ^1^ Department of Urology Cedars‐Sinai Medical Center Los Angeles California USA; ^2^ Department of Computational Biomedicine Cedars‐Sinai Medical Center Los Angeles California USA; ^3^ Department of Pathology University of Illinois Chicago Chicago Illinois USA; ^4^ School of Biological Sciences Seoul National University Seoul Republic of Korea; ^5^ Samuel‐Oschin Comprehensive Cancer Institute Cedars‐Sinai Medical Center Los Angeles California USA

**Keywords:** extracellular vesicles, therapeutic targets, pan‐cancer, proteomics, receptor kinases

## Abstract

Extracellular vesicles (EVs) play an important role in cancer progression and metastasis. The increasing clinical proteomics data provides an opportunity to uncover new biomarkers and therapeutic targets in cancer specific EVs. Here, we present an integrated data analysis approach that leverages a comprehensive protein catalog from EVs, blood plasma, and the cancer cell surface to identify EV‐associated proteins and evaluate their potential as therapeutic targets in cancer cells. Quantitative proteomics data from 12 cancer types (*n* = 2,272) identified 3,250 proteins that are commonly found in EV studies and EV quantity data. Receptor kinases (RKs) are crucial in cancer signalling pathways and have been widely studied targets. Our analysis focused on identifying RKs with significant high expression in cancer types that were also detected in EVs. Notably, PTK7 emerged as a significant target with high expression or survival association in seven independent cancer cohorts. We then explored the druggability of the RKs. Our analysis of drug repurposing network and RNAi screening suggested PTK7 as a potential therapeutic target with no FDA approved drugs in cancer. Overall, this integrative analysis proposes a framework to prioritize EV‐associated candidates for downstream therapeutic investigation in cancer.

## 1 Introduction

Cancer research has increasingly highlighted the importance of integrating diverse data sources to better understand tumour biology and identify therapeutic targets. The Clinical Proteomic Tumour Analysis Consortium (CPTAC) has been pivotal in providing proteogenomic datasets through the Proteomic Data Commons (PDC) (Thangudu et al. 2020), the National Cancer Institute's (NCI) largest public repository of proteogenomic comprehensive tumour datasets. Recent trends in proteomic research have also driven towards comprehensive analysis through integration and comparison of various data beyond transcriptomics (Li et al. 2023). Recent proteomic pan‐cancer studies revealed changes in protein acetylation and phosphorylation patterns (Geffen et al. 2023) and focused on comprehensive analysis in understanding functional status of oncogenic drivers (Li et al. 2023).

Extracellular vesicles (EVs) have emerged as critical players in cancer biology, serving as nanosized carriers of molecules that facilitate intercellular communication. Due to their ability to encapsulate molecules, their significance has drawn attention in cancer research as potential carriers of protein biomarkers (Zaborowski et al. 2015) or therapeutic targets (Kamerkar et al. 2017, Szabo et al. 2017). These EVs can carry a variety of molecules, but receptor kinases (RKs) are critical in developing therapeutic strategies because they play an important role in coordinating cell communication and altering signalling pathways, including cell proliferation, differentiation, and metabolic pathways in cancer cells (Stommel et al. 2007; Van Der Mijn et al. 2014; Zanetti‐Domingues et al. 2020). For example, EGFR is one of the widely studied targets owing to its significance and the availability of targeted therapeutic interventions. Recent research, including a notable review article that focuses on EGFR's presence within EVs, has yielded valuable insights into the implications of EGFR encapsulation within EVs (Zanetti‐Domingues et al. 2020). Notably, various other types RKs can also be packaged within EVs, exerting profound effects on neighbouring cells, which can alter the tumour microenvironment, impede signalling pathways, and potentially inhibit metastasis (Teixeira et al. 2023). In addition, the RKs contained in EVs have been reported to correspond to the phosphorylation profile of the tumour cells, suggesting that EV‐associated RKs reflect the molecular landscape and can provide insight into therapeutically relevant signalling pathways of cancer cells (Van Der Mijn et al. 2014). Another study found that by combining a TGF‐β RK inhibitor with an EV secretion inhibitor at conventional subtherapeutic doses, TGF‐β signalling could be normalized in breast cancer (Teixeira et al. 2023). Therefore, we decided to further investigate RKs in the context of EVs as a potential therapeutic platform. By doing so, we hoped to identify kinase inhibitors that may be suitable for drug repurposing to treat different types of cancer and RKs that show high potential for targeted drug development.

Moving beyond the identification of individual molecules within EVs, this study adopts a comprehensive approach. By integrating pan‐cancer proteomic profiles with a comprehensive protein catalog of EV cargo and cell surface, and clinical information, we aim to identify EV‐associated proteins as a starting point for prioritizing clinically relevant cancer targets and to evaluate their potential as diagnostic biomarkers and therapeutic targets. Leveraging this integrative strategy, we specifically focused on RKs detected in EVs that are therapeutically targeted in the cancer cell across various cancer types. This multi‐faceted approach may facilitate the identification of novel EV‐associated targets across cancers.

## 2 Methods

### 2.1 Pan‐Cancer Proteome Data Analysis

We collected proteome data from a total of 1,060 donors in 13 cohorts of 12 cancer types available in Proteomics Data Commons (PDC) (https://pdc.cancer.gov, version of December 21st in 2021) (Thangudu et al. 2020) that satisfied the following three inclusion criteria: (1) availability of proteome data for individual patients; (2) at least 20 patients per dataset to ensure statistical power; and (3) utilization of uniform data collection protocols, such as consistent sample preparation and MS analysis methods, to ensure data comparability. Using the MS/MS data, for each dataset, we first used the PE‐MMR tool (http://omics.pnl.gov/software/PEMMR.php) (Adusumilli and Mallick 2017) for precursor mass correction and refinement. The resulting MS/MS data in the mgf format were then searched using MS‐GF+ search engine (v.2019.07.03) (Kim and Pevzner 2014) against the unified UniProt Human reference database (2020_05 release) with 179 contaminants (e.g., trypsin, keratin) in the following settings: precursor mass tolerance of 10 ppm, semi‐tryptic search, static modification of carbamidomethylation (+57.021460 Da) on cysteine residues, and variable modification of oxidation (+15.994920 Da) on methionine residues. Peptide spectrum matches (PSMs) were identified with a false discovery rate (FDR) of 1% using the target‐decoy method. For quantification of these PSMs, we deisotoped their reporter ion intensities and carried out quantile normalization for the deisotoped intensities across the channels in each TMT or iTRAQ set to remove channel‐dependent variations (Channel of ‘Normalization of MS/MS data’ in Figure S1). We then calculated the relative abundances of the PSMs by normalizing the resulting intensities with the following intensities of the internal references in the individual TMT or iTRAQ datasets: 1) the universal reference generated by pooling all the samples used in 12 datasets and 2) the median intensities of the paired normal tissues adjacent to tumours in EOGC. We then generated a vector including the relative abundances of all PSMs from each TMT or iTRAQ set and then performed quantile normalization again on *n* vectors from *n* TMT or iTRAQ sets to remove systematic variations across the TMT or iTRAQ sets (TMT or iTRAQ sets of ‘Normalization of MS/MS data’ in Figure S1). To ensure high quantitative accuracy and minimize the impact of precursor interference, a Precursor Ion Purity (PIP) threshold of >70% was applied (Figure S2). This cutoff was chosen based on its proven efficacy in identifying reliable differentially expressed proteins (DEPs) while filtering out approximately 25% of likely false positives (Li et al. 2014). This threshold is consistent with standard pipelines for multiplexed proteomic analysis (Hyeon et al. 2023; Keshishian et al. 2017). Next, we merged the normalized relative intensities into an *m* x *k* matrix for *m* PSMs and *k* samples after selecting reliable PSMs with PIP > 70% (Generation of peptide abundance table’ in Figure S1).

Protein grouping was performed using a bipartite‐graph‐based parsimony algorithm to resolve shared peptides (Mun et al. 2019; Dost et al. 2012). Proteins within each connected component were mapped to UniProt identifier, and isoforms/protein groups collapsed to a single representative protein entity (UniprotKB accession number (AC) based) to mitigate ambiguity from shared peptides. For quantification, peptide intensities were summarized as the median across non‐redundant peptides, requiring at least two peptides per entity. And, then we quantile‐normalized the resulting relative protein abundances after keeping only the proteins whose relative abundances were available in more than 70% of samples to ensure statistical power in the subsequent analyses (Figure S1). Table S1 shows the numbers of proteins.

### 2.2 Clinical Data Curation

Clinical data of the pan‐cancer proteome cohorts were downloaded from the PDC data portal to create a combined dataset of clinicopathological characteristics of the patients. We processed 13 datasets across 12 different cancer types. Each cohort's clinical profile, including age, sex, race, body mass index (BMI), and pathologic stage, is detailed in Table S2. BMI information is available for 9 cohorts, while survival data is provided for 11 cohorts. Racial data is delineated across 12 cohorts and categorized into 6 groups: White, Black, Asian, Hispanic, American Indian, and Unknown. Individuals of European descent, excluding those of Hispanic ethnicity, were classified as White. BMI was either directly provided by the original dataset or calculated using the authors' provided height and weight measurements (calculated BMI = [weight]/[height]^2^). Tumor stage information, originally ranging from stage 1 to 4, was reclassified into two groups for analysis purposes: stages 1 and 2 combined as one group, and stages 3 and 4 combined as the second group.

### 2.3 Collection of EV Molecular Profiles

The EV molecular profiles include the detected experiment count data and quantity data obtained from each different data resource. The EV data, specifically the experiment count data detected for each EV, was collected from Vesiclepedia v4.1 (Pathan et al. 2019). The EV quantity data was compiled from EV data of samples excluding plasma samples as described in the study of Hoshino et al. (2020). In both EV datasets, we summarized data focusing on EVs associated with cancer types of pan‐cancer proteome data we compiled. In addition, experiment counts from Vesiclepedia were used only as a literature‐consensus indicator of proteins commonly reported in EV studies. To reduce reliance on a single repository, we additionally cross‐checked our RK candidates using ExoCarta (Keerthikumar et al. 2016) and the HPA secretome (Uhlén et al. 2019).

### 2.4 Summary of Human Plasma Proteome

The human plasma dataset, sourced from the Human Protein Atlas (Uhlén et al. 2019), offers estimated concentrations of 4072 proteins identified in human plasma through mass spectrometry analysis. This dataset includes the estimated concentration of proteins that are actively released into the plasma, locally secreted proteins, or intracellular proteins that have entered the bloodstream.

### 2.5 Selection of Cancer Surfaceome

The cell‐surfaceome data used in this study was originally obtained from Zhongyi Hu et al. (2021), which weighted various information to provide the final Genes Encoding cell‐Surface Proteins (GESP) score values. This score is positively correlated with the likelihood of a gene encoding a surface protein, with higher GESP scores indicating a greater probability. We finally selected 3,567 human GESP genes based on their GESP scores and associated probabilities. Additionally, to assess the extracellular accessibility of the candidates RKs, we investigated topology annotation. We extracted transmembrane (TM) domain counts and signal peptide information from UniProt and the Cell Surface Protein Atlas (CSPA) (Bausch‐Fluck et al. 2015; Bausch‐Fluck et al. 2018), which provides experimental evidence for cell‐surface localization via surfaceome proteomics.

### 2.6 Dimension Reduction and Functional Association Analysis of Pan‐Cancer Proteome

For integrated/global visualizations (PCA/UMAP), we restricted the 5,471 proteins detected across all 13 cohorts (*n* = 1475 individuals), imputed remaining missing values within each cohort using kNN imputation (*k* = 10) (Troyanskaya et al. 2001), and applied ComBat (sva, v3.50.0) (Leek et al. 2012) to mitigate cohort‐level batch effects. PCA and UMAP were computed using the batch‐corrected matrix. As a sensitivity analysis, we repeated PCA using left‐censored MNAR imputation (MSnbase, v2.28.1) (Gatto and Lilley 2012), which produced similar embeddings. Diagnostic PCA plots (before/after batch correction and kNN vs MNAR) are provided in Figure S3. Subsequently, we performed PCA using the PCAtools R package (v4.2.2) (Blighe and Lun 2021). The UMAP analysis by cancer type was conducted using the umap package (UMAP 2018) with a fixed random seed (seed = 42), Euclidean distance metric, n_neighbors = 15, and min_dist = 0.1. Sensitivity analyses were conducted by varying n_neighbors (10,15,30) and min_dist (0.05, 0.1, 0.3), and the resulting embeddings are provided in Figure S4. We selected the top 8 principal components of PCA results by using the Scree plot. In this manner, 3250 common EV‐associated proteins were also employed for UMAP analysis by selecting the top 8 principal components for each cancer type (Figure S5). The protein classes of common and cancer‐specific genes were categorized using the Panther Classification System from the PANTHER database (Thomas et al. 2022). We curated a list of common and cancer‐specific genes in various cancers and classified the genes into protein classes. Additionally, using Gprofiler2 (v 0.2.3) (Kolberg et al. 2020), we conducted functional enrichment analysis against the Gene Ontology (GO) Cellular Component to identify the localization of the proteins enriched in EVs compared to the proteins rarely detected in EVs. We filtered the top 20 enriched cellular localization with *p*‐value < 0.05 (Figure S6).

### 2.7 The Cancer‐to‐Cancer Association Heatmap

We represented the degree of EV proteins sharing across cancer types by calculating z‐scores of the Jaccard Index (Jaccard similarity coefficients) using a custom R function. To visualize this, we employed the ComplexHeatmap R package (v. 2.18.0) (Gu et al. 2016) to generate a detailed heatmap, facilitating the clustering and comparison of protein sharing across the different cancer types.

### 2.8 Identifying Clinically Targetable RKs

To search for therapeutic targets of RKs, we utilized information on RKs from the HGNC (HUGO Gene Nomenclature Committee, https://www.genenames.org/; Seal et al. 2023) and focused on 70 RKs. We employed two approaches to obtain candidate RK as therapeutic drug targets. The initial approach involved scrutinizing protein presence using Vesiclepedia (Pathan et al. 2019) and overlapping with the surfaceome list from a published article (Hu et al. 2021). Simultaneously, the second approach focused on protein quantity data to discern differential expression between cancer and normal samples. For identifying differentially expressed proteins, we employed the Wilcoxon test to calculate *p*‐value and applied the Benjamini–Hochberg procedure to adjust for multiple testing across all proteins tested within each cohort (BH‐FDR). To determine appropriate log2 fold‐change cutoffs, we generated permuted datasets and derived quantiles to assess the variability of log2 fold‐change values. We used 95% quantile as log2 fold‐change cutoff in each protein data of cohorts to do the analysis of differential expression (Shin et al. 2017). The number of proteins tested per cohort and the BH‐FDR–adjusted results for RKs are provided in **Supplementary File** **1**. Volcano plots and *p*‐value histograms for representative cohorts are shown in Figures S7 and S8. In cohorts where normal samples were unavailable (BRCA, EOGC and PCKU), survival analysis using multivariable Cox models was conducted to complement the identification of candidates. For each cohort, continuous protein abundance was used as the predictor, adjusting for age, sex, and stage. A total of 26 RK candidates were prioritized by considering these survival results in conjunction with the DEPs in other cohorts. To investigate the survival effects of these 26 candidates across cancer cohorts, we employed the same multivariable Cox models and displayed the results in a forest plot, highlighting proteins with HR > 1 and *p* < 0.05. Kaplan–Meier (KM) survival plots were used for visualization and were generated by dichotomizing protein abundance using the cutpointr package (v1.1.2) (Thiele and Hirschfeld 2020). Optimal cutoff values were determined via the Youden index, and the robustness of the selected cutoff values was assessed by bootstrap resampling(boot_runs = 1000) (Figure S9). Visualization was performed using the survival (v3.4) and survminer (v0.4.9) packages ([cited 2021 Jan 29]).

### 2.9 Reconstruction of FDA Approved Drugs and Their Targets Association Network

We utilized drugst.one (v1.2.0) (Maier et al. 2024) to construct a drug repurposing network. Significant RKs by cancer types served as input for the search algorithms. NeDRex (v2.21.0) (Sadegh et al. 2021) was employed to search for protein‐drug interaction. Subsequently, following the acquisition of a drug list corresponding to each target protein, a meticulous cross‐referencing process through searching in FDA approved drug list, was undertaken to align individual drugs with the current FDA‐approved treatments for each cancer types(https://www.fda.gov/).

Using the STRING database, a comprehensive resource for protein‐protein interaction data (Szklarczyk et al. 2023), we reconstructed a network model of 26 RKs. To assess the known associations between these RKs and specific cancer types, we leveraged target–disease association evidence from the Open Targets Platforms (Ochoa et al. 2023). Open Targets association scores were calculated by taking the harmonic sum of scores from various data sources, each weighted according to its relevance, reflecting the cumulative evidence across multiple datasets; germline and rare disease genetics (GWAS Catalog, ClinGen, ClinVar), somatic mutations in cancer (Cancer Genome Interpreter, IntOgen), RNA expression (Expression Atlas), drugs and clinical indications (ChEMBL, clinical trials), affected pathways/systems biology (Reactome, STRING, IntAct), and literature evidence (Europe PMC) (Ochoa et al. 2023). To quantify target novelty, we implemented a rank‐based novelty metric within the RK universe. Specifically, for each RK, we computed the percentile rank of its Open Targets association score across the full RK set, and defined novelty as: Novelty score = 1 − percentile rank (association score). The novelty scores and absolute association scores for the 26 prioritized RK candidates are provided in Table S7, and novelty scores for the full RK set are provided as separate **Supplementary data file 2**.

### 2.10 Drug Sensitivity and Druggability Assessment Using DepMap

We investigated the drugs associated with our candidate RK targets using drug sensitivity data. We found drug sensitivity data for 40 out of the 47 drugs from the “PRISM Primary Repurposing DepMap Public 24Q2” dataset (Repurposing_Public_24Q2_LFC_COLLAPSED.csv) on DepMap (Corsello et al. 2020). Specifically, we mapped DepMap lineage annotations to our cancer‐type labels (Table S8), we used the final processed PRISM log2 fold‐change viability values (LFC), which were median‐collapsed across QC‐passing replicates from the single‐dose (2.5 µM) screen and represent the change in estimated cell abundance in drug‐treated wells relative to DMSO controls. For empowering findings about vulnerabilities of cancer cells when targeting specific RKs, we downloaded expression data (DepMap 23Q4 Public) and the gene effect scores of RNAi (Achilles+DRIVE+Marcotte, DEMETER2) for each cancer cell line from DepMap (Tsherniak et al. 2017; Mcfarland et al. 2018). To assess the significance of the RNAi gene effect score of PTK7, we first randomly selected 20% of genes with RNAi gene effect scores from DepMap and computed the average of the scores. A null distribution representing random effect scores was created using gene effect scores of 20% random genes and compared to the actual PTK7 RNAi effect score distribution. Also, when examining the RNAi gene effect scores of PTK7 across different lineages, we focused on those lineages present in the cancer types included in our pan‐cancer proteome.

## 3 Results

### 3.1 Investigation Into the Molecular Profile of Proteomic Data

**FIGURE 1:**
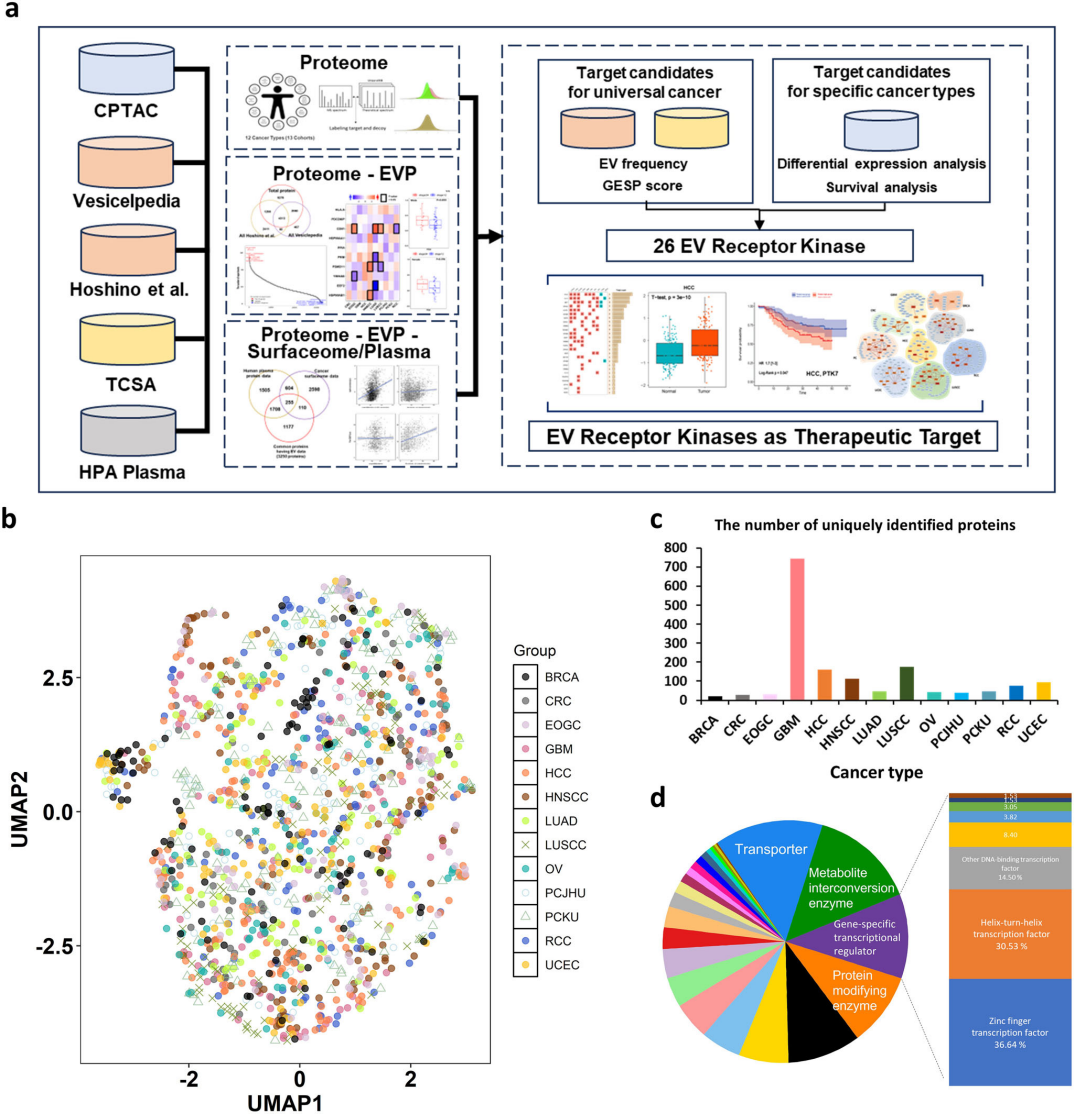
Overview of the analytical framework and the characteristics of proteomic molecular profiles. (a) Overview of finding EV associated surface protein markers. (b) UMAP by samples of integrated proteome data. BRCA, breast cancer; CRC, colon cancer; EOGC, early onset gastric cancer; GBM, Glioblastoma; HCC, Hepatocellular Carcinoma; HNSCC, Head and neck squamous cell carcinoma; LUAD, Lung adenocarcinoma; LUSCC, Lung squamous cell carcinoma; OV, Ovarian cancer; PCJHU, Pancreatic ductal adenocarcinoma from Johns Hopkins University; PCKU, Pancreatic ductal adenocarcinoma from Korea University; RCC, Renal Cell Carcinoma; UCEC, Uterine corpus endometrial carcinoma. (c) The number of proteins detected in only one cancer by cancer type. (d) Proportions by functional molecular class of proteins were detected in only one cancer by cancer type.

We performed an extensive integration of pan‐cancer proteomics data with molecular profiles from EVs, cancer cell surfaces, and human plasma, aiming to characterize highly detectable proteins that serve as potential therapeutic targets. The pan‐cancer proteome data was obtained from Proteome Data Commons (PDC) (Thangudu et al. 2020). This is a large collection of mass spectrometry‐based proteomics profiles from pan‐cancer patient tissues and processed by the in‐house method (see Methods). It encompasses a large collection of proteome profiles from 13 cohorts representing 12 types of cancer (BRCA, breast cancer; CRC, colon cancer; EOGC, early onset gastric cancer; GBM, Glioblastoma; HCC, Hepatocellular Carcinoma; HNSCC, Head and neck squamous cell carcinoma; LUAD, Lung adenocarcinoma; LUSCC, Lung squamous cell carcinoma; OV, Ovarian cancer; PCJHU, Pancreatic ductal adenocarcinoma from Johns Hopkins University; PCKU, Pancreatic ductal adenocarcinoma from Korea University; RCC, Renal Cell Carcinoma; UCEC, Uterine corpus endometrial carcinoma). The dataset includes a total of 2,272 samples consisting of 1,475 tumors and 797 normal samples (Table S1). EV quantity was obtained from Hoshino et al. (Hoshino et al. 2020) and Vesiclepedia data were used to indicate proteins commonly reported in EV studies (Pathan et al. 2019). The estimated plasma concentration of proteins was obtained from the Human Protein Atlas (Uhlén et al. 2019) and the Genes Encoding cell‐Surface Proteins (GESP) score values from The Cancer Surfaceome Atlas (TCSA) (Hu et al. 2021). Subsequently, we utilized the combined dataset to identify candidate target molecules. We examined and outlined the relationships among various datasets. To identify EV RK candidates as therapeutic targets, we used two filters; one based on proteins commonly reported in EV studies and GESP score across cancers, and another based on differential expressed proteins or high‐risk proteins in specific cancer types. We then focused on 26 EV RK markers that emerged as common candidates across these two filters. Finally, we validated EV RK markers through multiple analysis including survival analysis and evaluation of druggability (Figure 1a). We investigated the global molecular structure of the integrated proteomic dataset using UMAP (Figure 1b). For the integrated dataset, samples from different cohorts were broadly intermixed in the UMAP space. Although some cancer types showed modest tendencies to occupy specific regions, the overall embedding did not exhibit strong cohort‐specific clustering.

When investigating the molecular classes of proteins detected across 13 cancer cohorts or those specific to one cancer cohort, as shown in Table S3, we found that metabolite interconversion enzymes were the most abundant among common proteins, highlighting metabolic changes commonly observed in cancer (Figure S10). On the other hand, in uniquely detected proteins specific to one cancer, GBM was found to have the highest number of unique proteins (Figure 1c). Uniquely detected proteins revealed the predominance of transporter proteins, with gene‐specific transcriptional regulators also prominently represented (Figure 1d). However, this is because unique proteins of GBM were mostly associated with transporter groups (Table S4).

### 3.2 Comparison of Proteomic Data With EV‐Associated Proteins

**FIGURE 2:**
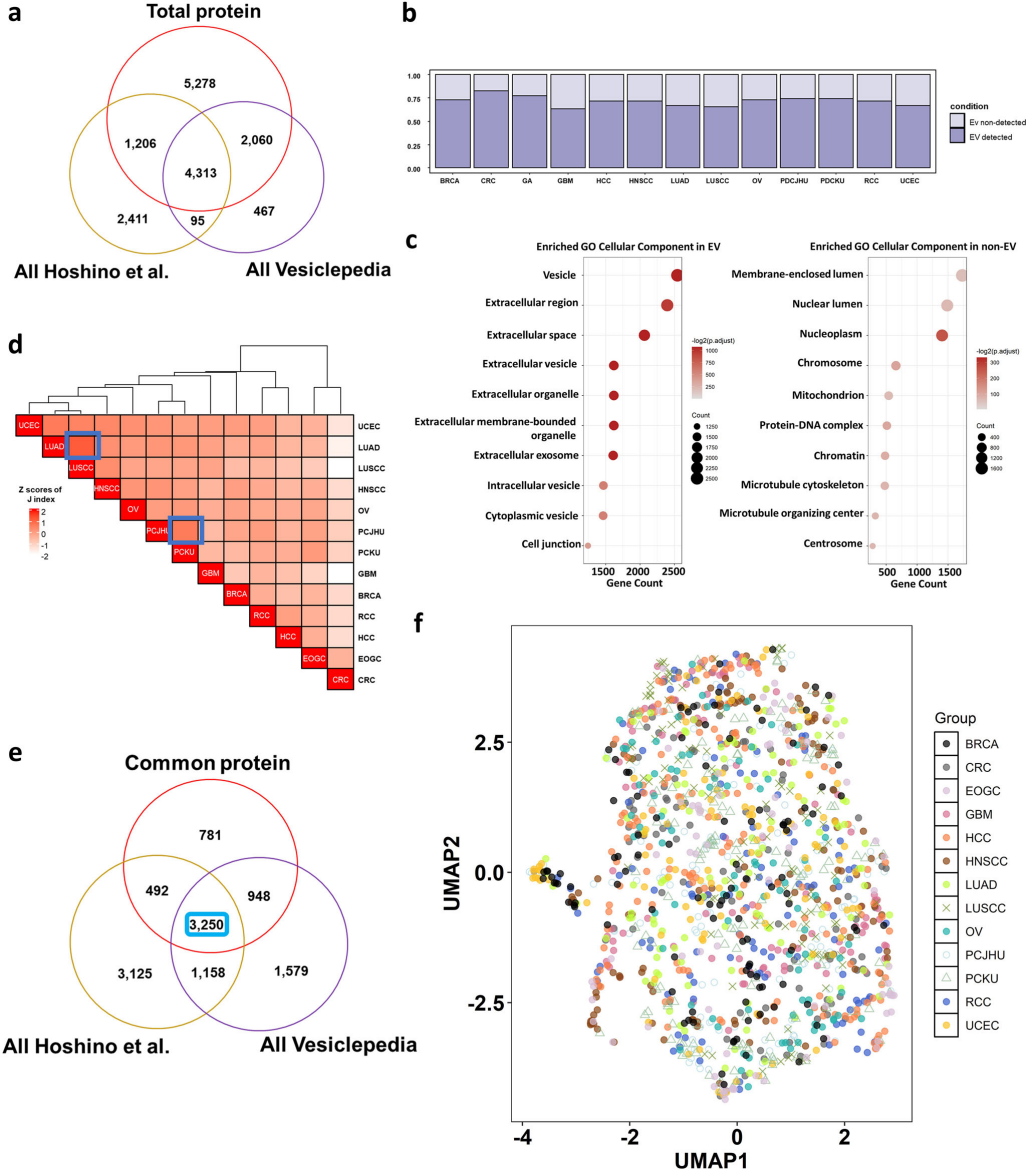
The characteristics between Proteomic and EV data. (a) Venn diagram illustrating the overlap between EV datasets and protein data. (b) Proportions of EV‐associated proteins among total proteins detected in cancer types. (c) Gene Ontology (GO) enrichment analysis of each EV‐associated protein and non‐EV protein for Cellular Components. The dot plots show the specific selected cellular components in the top 20 significant terms for EV‐associated proteins (left) and non‐EV proteins (right) (based on adjusted *p*‐value). (d) Heatmap showing Z‐scores of Jaccard Index (Jaccard similarity coefficients) for sharing EV‐associated proteins between cohorts. (e) Venn diagram illustrating the overlap between EV datasets and the proteins commonly detected across cancer types. (f) UMAP by samples of common EV proteome data.

We used two different resources for EV molecular profiles; the EV molecular quantities from Hoshino et al. (2020) and the number of detections from cancer EV experiments at which each protein was observed from Vesiclepedia (Pathan et al. 2019). We curated EV‐associated protein quantities and the number of detections of the EV‐associated proteins and compared these data with our proteomic data. Based on our proteomic data, we found 4,313 EV‐associated proteins of our total 12,857 proteins (Figure 2a). Figure 2b shows the proportions of EV‐associated proteins to the number of total proteins detected by cancer types. Approximately 70% of detected proteins were also found in EVs, with GBM having the lowest overlap. EV‐associated proteins were linked to GO terms for EVs, while non‐EV proteins were associated with intracellular locations like the nucleus (Figure 2c). In Figure 2d, relationships between cancer types were depicted as the Z‐scores of the Jaccard similarity index representing the degree of EV‐associated protein sharing between different cohorts. CRC revealed to share fewer EV‐associated proteins with other cancer types. Additionally, a high level of EV‐associated protein sharing was observed between LUSCC and LUAD, as well as between PCJHU and PCKU, which are pairs of cancers originating from the same organ. There are 5,471 proteins shared across all 13 cohorts, with 3,250 of these proteins overlapping in two EV data (Figure 2e). We compared the characteristics of 3,250 common proteins from EV and proteome datasets using dimensionality reduction. Most cancer types exhibited a similar spatial distribution on the UMAP, with minimal separation by tissue of origin. This UMAP also was not clearly separated and instead showed extensive overlap (Figure 2f).

### 3.3 The Clinical Profile Characteristics of EV‐Associated Proteins From Proteomic Data

**FIGURE 3:**
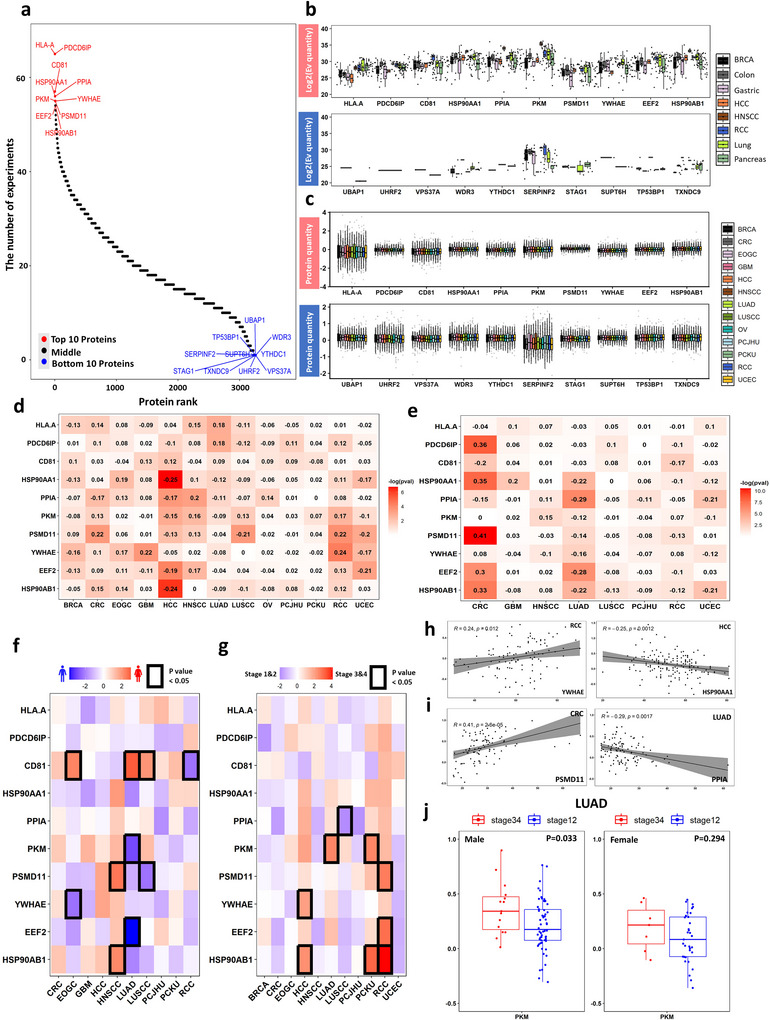
Clinical characteristics of common EV‐associated proteins in proteome data. (a) Distribution of reporting frequency across EV studies of common proteins from Vesiclepedia. The top 10 most frequently reported proteins are highlighted in red, and the bottom 10 are in blue. (b) Expression levels of the top and bottom 10 EV proteins from EV quantity data of Hoshino et al. (c) Expression levels of the top and bottom 10 EV proteins from CPTAC integrated proteome data. (d, e). Heatmap showing the correlation between clinical variables and top 10 EV‐associated proteins. The numbers in boxes are the Pearson correlation coefficient (*r*) for Age (d) and BMI (e). (f, g) Heatmaps showing the differences by sex and tumor stage for the top 10 EV‐associated proteins. T‐scores show comparing Male vs. Female (f) and Early (Stage 1–2) vs. Advanced (Stage 3–4) tumor stages (g). Red indicates higher expression in females or advanced stages; blue indicates higher expression in males or early stages. Statistically significant proteins (*p* < 0.05) are highlighted within boxes. (h) Correlation plots of proteins with high (YWHAE of RCC) or low (HSP90AA1 of HCC) correlation between Age and EV‐associated proteins. (i) Correlation plots of proteins with high (PSMD11 of CRC) or low (PPIA of LUAD) correlation between BMI and EV‐associated proteins. (j) Expression levels of PKM between Stage1‐2 and Stage 3–4 group by sex in LUAD.

To further explore the characteristics, we selected the top 10 and bottom 10 proteins based on how commonly they were reported across cancer EV studies in Vesiclepedia among the 3,250 common proteins (Figure 3a), excluding immunoglobulins, which typically express high quantities. From the box plots of Figure 3b, we observed that proteins with higher reporting frequency in Vesiclepedia (top 10 proteins) were positively associated with the number of samples having available data from EV quantity data, while the bottom 10 proteins had fewer. However, in our tissue protein data, unlike the EV quantity data, the sample availability was similar for both the top and bottom 10 EV‐associated proteins (Figure 3c). With these top and bottom 10 EV‐associated proteins, we explored the correlation between the levels of EV‐associated proteins with associated proteome data with clinical characteristics including age, BMI, sex, and stage. In Figure 3d,e, the heatmaps show the correlation between top 10 EV‐associated proteins and each age and BMI. Correlation analysis revealed that HSP90AA1 and HSP90AB1 were negatively correlated with age in HCC, while YWHAE was positively correlated with age in RCC (Figure 3d,h). The significant correlations with BMI were also observed, particularly in colon cancer where PDCD6IP, HSP90AA1, PSMD11, EEF2, and HSP90AB1 showed positive correlations. In contrast, several proteins, including PPIA, displayed negative correlations in LUAD (Figure 3e,i). The correlations between top 10 EV‐associated protein levels and sex or stage are shown in Figure 3f,g. CD81 was more highly expressed in females with EOGC, LUAD, and LUSCC, but higher in males with RCC (Figure 3f). HSP90AB1 had significantly higher expression in the group of stages 3 and 4 in HCC, PCKU, and RCC (Figure 3g). Interestingly, PKM was significantly correlated with both sex and stage in LUAD, showing higher expression in males and in stages 3 and 4, respectively. Notably, when analyzed by sex, PKM expression was significantly higher in stages 3 and 4 only in males (Figure 3j).

### 3.4 Comparison of each Human Plasma and Cancer Surfaceome Data With EV Data

**FIGURE 4:**
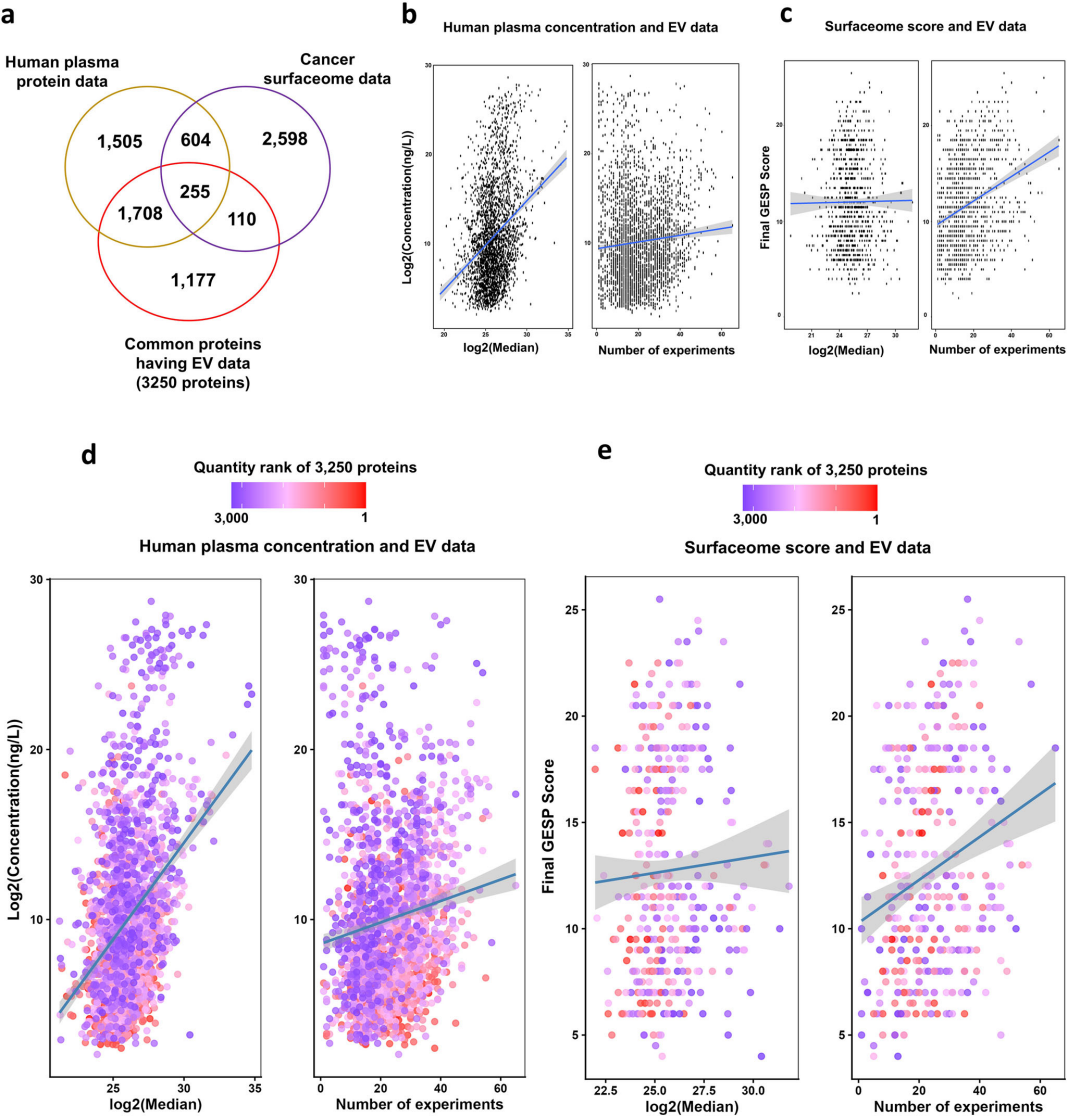
The characteristics of human plasma and cancer surfaceome data by EV data. (a) Venn diagram comparing 3,250 common proteins with Human plasma protein data and cancer surfaceome data. (b) Scatter plots showing relationship between EV data (EV quantity and experiment counts from Vesiclepedia) and Human plasma data. (c) Scatter plots showing relationship between EV data (EV quantity and experiment counts from Vesiclepedia) and cancer surfaceome data. (d) Scatter plots of 1,963 proteins between EV and Human plasma data presenting quantity of common proteins. (e) Scatter plots of 365 proteins between EV and cancer surfaceome data presenting quantity of common proteins.

We found 1,963 proteins overlapping with human plasma data and 365 proteins with cancer surfaceome data among the common 3,250 EV‐associated proteins across the 13 cancer cohorts (Figure 4a). We examined the relationship between plasma concentrations from HPA and cancer surface data from Zhongyi Hu et al. (2021) (Figure 4b–e). Although similar patterns almost emerged in both EV datasets concerning their association with human plasma data or cancer surfaceome data (Figure 4b), a positive correlation in cancer surfaceome data was not observed with the EV quantity data (Figure 4c). Additionally, scatter plots of the overlapped proteins showed no clear relationship between protein quantity rank and either plasma concentration or GESP score (Figure 4d,e).

### 3.5 **The Search for** RKs **as Therapeutic Drug Targets With Clinical Protein Data**

**FIGURE 5:**
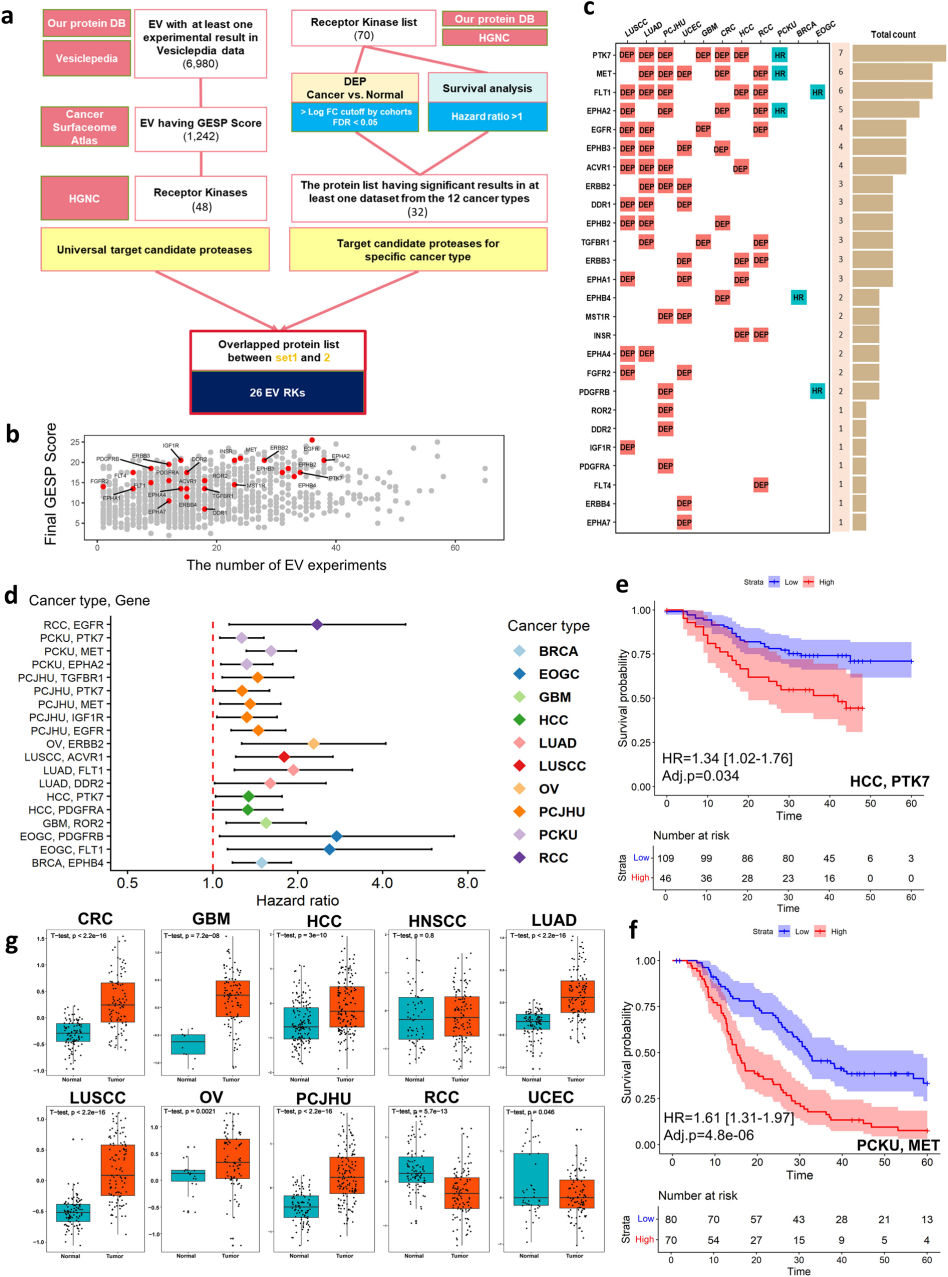
Selection of candidate RKs for therapeutic drug targets. (a) A scheme of selection process for EV‐associated RKs. (b) Scatter plot between the number of EV experiments and GESP score of common proteins. (c) Summary of Significance for 26 candidate RKs by cancer type. (d) Forest plot of hazard ratios for RKs. HRs were calculated using multivariable Cox proportional hazards models adjusting for age, sex, and stage. Only findings with HR > 1 and covariate‐adjusted *p* < 0.05 are shown. (e, f) Kaplan‐Meier survival plots of low versus high protein expression groups for PTK7 in HCC (e) and MET in PCKU (f). (g) Expression levels of PTK7 in normal (blue) and tumor (red) samples by cancer type.

To select candidate RKs, we followed our two approaches as depicted in Figure 5a. First, we selected EV‐associated proteins commonly reported in EV studies (Vesiclepedia) and with a GESP score of surfaceome data. Subsequently, by narrowing the scope to RKs, we obtained a list of 48 universal candidate targets regardless of cancer type. In our second approach, we obtained a list of 32 proteins showing significant results in at least one dataset from the 13 cancer cohorts. Comparing the protein lists from set 1 and 2, we identified 26 overlapping RKs. When examining the distribution of EV and GESP scores in 3,250 common proteins possessing both EV and GESP score data, 26 candidate RKs appear to be situated toward the higher range (Figure 5b). Figure 5c describes the count of cohorts showing the significance of each 26‐candidate RKs in the second approach. Each 26‐candidate RK showed significance in at least one cohort. Notably, PTK7 exhibited significance in 7 cancer cohorts (Figure 5c, Table S5). We cross‐referenced the 26 RK candidates in ExoCarta and the HPA secretome and annotated membrane topology to support extracellular accessibility (Table S6). Among the 26 candidates, all were annotated to possess at least one transmembrane domain based on UniProt topology information, and 22 were supported as cell‐surface proteins by CSPA, supporting their potential surface accessibility. To further investigate, we proceeded to evaluate 26 RKs using clinical data. Higher protein abundance of several candidate RKs was associated with worse survival (HR > 1) in multiple cancer cohorts (Figure 5d). Kaplan–Meier curves further illustrated improved survival in the low‐expression group compared with the high‐expression group for these candidates (Figure S11). Notably, patients with low expression levels of PTK7 and MET showed higher survival rates (Figure 5e,f). Furthermore, PTK7, consistently highlighted across cancer cohorts, showed higher abundance in several tumor groups than in normal groups (Figure 5g).

### 3.6 **The Association Between Selected** RKs **and Therapeutic Drugs**

**FIGURE 6:**
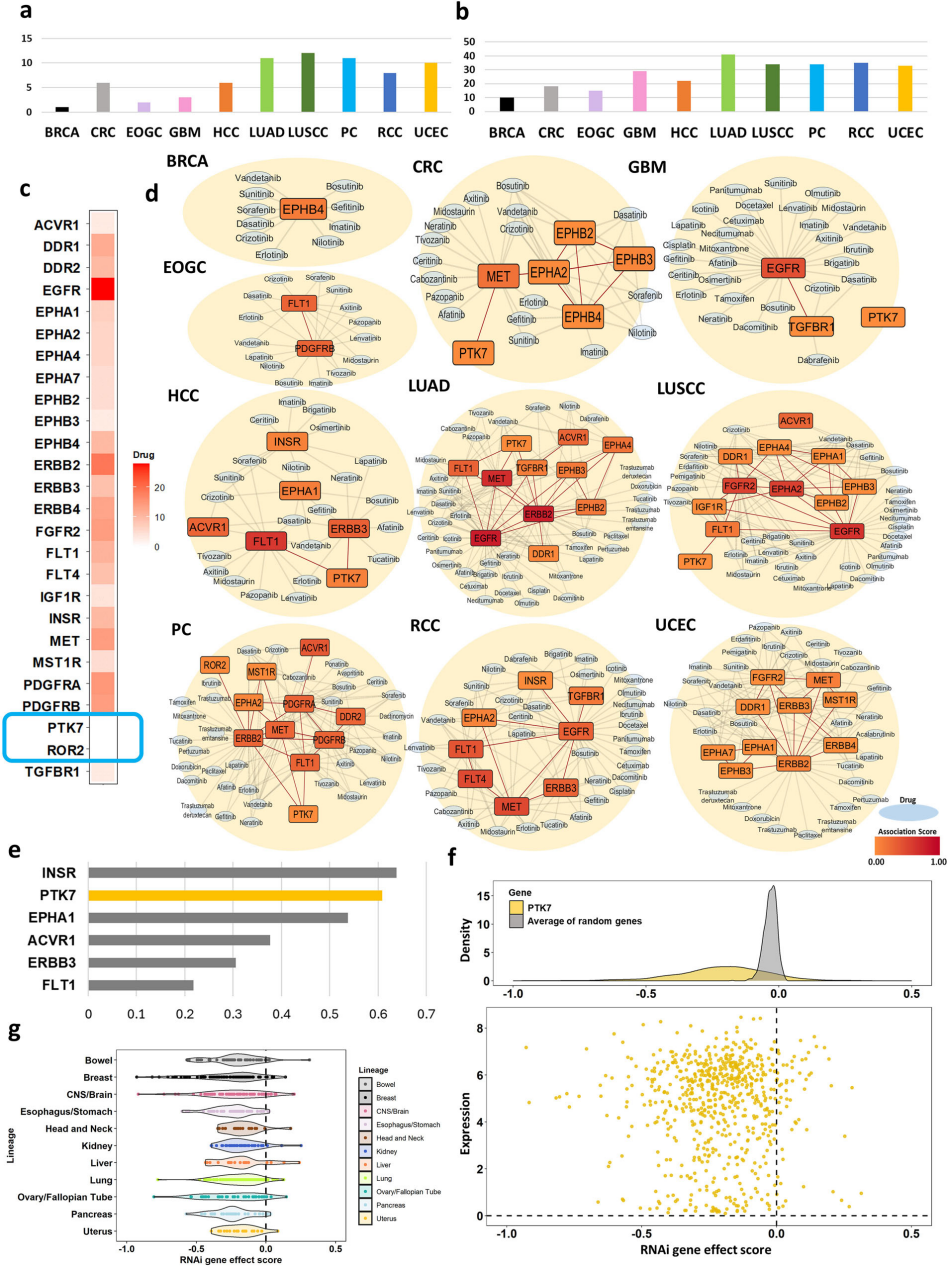
Association of 26 candidate RKs with therapeutic drugs. (a) The number of significant RKs by cancer types. (b) The number of drugs associated with significant RKs by cancer types. (c) Heatmap showing the number of drugs by 26 RKs. (d) Association network of significant RKs by cancer type with drugs. The rectangles represent RKs. The light blue ovals are drugs. The color of the rectangles (orange to red) indicates the association score with the cancer. (e) The novelty score (1−percentile rank (association score associated with HCC)) of significant RKs in HCC. (f) Density plot of RNAi gene effect scores of PTK7 and average RNAi gene effect scores of random genes (upper) and scatter plot of RNAi gene effect scores and expression of PTK7 (lower). (g) Violin plots of RNAi gene effect scores of PTK7 by lineages.

To assess the clinical potential of the identified markers, we examined therapeutic drugs targeting the 26 RKs, focusing on their interactions in different cancer types. Figure 6a shows the number of significant RKs found in our analysis by cancer type. LUSCC had the highest number of RKs and BRCA was the lowest. The number of drugs linked to significant RKs varied across cancer types, with LUAD reaching the highest count and BRCA the lowest (Figure 6b). Of the 26 common RKs, EGFR had the greatest number of drugs associated with it, followed by ERBB2. PTK7 and ROR2 did not have any associated drugs (Figure 6c). We visualized networks between the RKs and their associated drugs, which are FDA‐approved drugs for cancers, and showed the association scores of corresponding RK targets obtained from Open Targets Platforms (Ochoa et al. 2023) in different shades of red (Figure 6d). To further explore the associated drugs in our networks, we used drug sensitivity data from the DepMap database for evaluating their activity in cell lines. Dasatinib, Midostaurin, Ceritinib, Mitoxantrone, Docetaxel, and Doxorubicin were found across multiple cancer types in our network analysis. Notably, these drugs also exhibited consistently negative median LFC (log2 fold‐change viability) values, indicating reduced cancer cell viability upon treatment (Figure S12). We also computed the novelty score with the association score, and Figure 6e shows novelty scores of significant RKs in HCC. While the literature score of PTK7 associated with HCC was the third (Table S7), which is 0.77, the novelty score is the second highest. This indicates that PTK7 is a novel target that has been observed to be associated with HCC in several publications but has not yet been extensively covered in various data resources. To further validate PTK7 as a viable target, we analyzed RNAi gene effect scores from the DepMap database. In Figure 6f and Figure S13, we found that the scores for PTK7 were significantly more negative compared to a null distribution of randomly selected genes (*p*‐value < 2.2e‐16), indicating that PTK7 knockdown effectively inhibits cancer cell growth, reinforcing its potential as a therapeutic target. Not only in the total RNAi gene effect score data, but also in the lineage‐specific scores from DepMap, most RNAi gene effect scores of PTK7 were negative values, suggesting that it can be a target for various cancers (Figure 6g).

## 4 Discussion

This study represents a comprehensive effort to gather and curate diverse cancer proteomic data resources with EV protein catalogs to facilitate the discovery of clinically relevant cancer targets. In recent advancements in protein resources (Adusumilli and Mallick 2017; Bausch‐Fluck et al. 2018; Bausch‐Fluck et al. 2015), our study stands out by not only encompassing pan‐cancer proteome data from the PDC data portal but also incorporating additional datasets from the relevant publication. Given the potential of EV‐associated proteins to serve as reliable markers for cancer subtyping and identification (Hoshino et al. 2020), our protein dataset incorporates EV and cancer surfaceome data that can provide essential evidence for finding EV‐associated proteins for therapeutic target research. By linking proteomic profiles with EV and cancer surfaceome data, we prioritized RKs that are detected in EV studies and supported as actionable targets, with extracellular accessibility further validated by UniProt/CSPA topology annotations. This approach, using EV information to identify candidate targets, extends the translational potential of EV research and enables the prioritization of druggable candidates across multiple cancer types.

In proteome data, we revealed characteristics of common and unique proteins by investigating the molecular classes of these two groups across cohorts. Notably, the unique proteins of GBM were linked to transporter groups, related to neurotransmission pathways (Table S4). This highlights the potential for further investigations into specific proteins detected in individual cancer types to offer valuable insights into the underlying mechanisms of cancer development. Additionally, integrating protein and EV data revealed that EV‐associated proteins were enriched in extracellular compartments, emphasizing their key role in intercellular communication and tumor microenvironment modulation, while non‐EV proteins were linked to intracellular compartments like the nucleus, indicating distinct cellular functions. This underscores the importance of EV‐associated proteins in modulating the tumor microenvironment, reinforcing their relevance as intercellular communication mediators (Kalluri 2016; Hoshino et al. 2015), while simultaneously providing a window into the clinically relevant RKs that can be targeted for therapeutic intervention.

By combining proteomic and EV data, we identified correlations between specific EV‐associated proteins and clinical characteristics. Notably, the PKM protein exhibited significant correlations with both sex and stage in LUAD. PKM, crucial in glycolysis, has been linked to tumor growth, invasion, and metastasis by regulating glycolytic flux (Chen et al. 2020; Lu et al. 2016; Puckett et al. 2021). While PKM upregulation is consistently associated with poorer outcomes in lung adenocarcinoma, our finding of a correlation with sex requires further investigation to determine whether it reflects a sex‐specific impact on metabolic pathways in LUAD.

Finally, our study identified 26 RKs with therapeutic potential by integrating EV and clinical protein data across cancer cohorts. Exploration of clinical, drug, and RNA interference data further highlighted several candidates as promising therapeutic targets. Notably, the association with FDA‐approved drugs underscores the potential translational significance of candidates. These drugs are being used as anticancer agents, have been involved in clinical trials, or are being proposed as novel therapeutic strategies in combination with other drugs (Lamture et al. 2018; Jamshed et al. 2020; Kristeleit et al. 2021; Hsiao et al. 2019; Lue et al. 2021). For example, Doxorubicin was found in our network analysis for LUAD, PC, and UCEC, and showed a negative median LFC in cancer cell line data. Clinically, it has been used in NSCLC and has shown good effects in a phase I study of recurrent advanced endometrial cancer when combined with other drugs (Kristeleit et al. 2021).

Furthermore, PTK7, one of 26 RK candidates, showed high expression in various cancers and a high hazard ratio for PTK7 in HCC. In our study, although PTK7 has been studied across several cancers, our integrative framework highlights its potential relevance by jointly supporting its EV association, extracellular accessibility, and poor prognostic association in HCC. Recent studies highlight PTK7 as a promising target for therapeutic intervention showing its overexpression in various cancers, including triple‐negative breast cancer, non‐small cell lung cancer, and ovarian cancer (Cui et al. 2021; Jiang et al. 2020; Raivola et al. 2022; Dessaux et al. 2024). PTK7 has also been explored as a target for CAR T cell therapy in lung cancer (Jie et al. 2021) and as an antibody‐drug conjugate (ADC) target (Damelin et al. 2017). PTK7‐targeted ADCs (Maitland et al. 2021; Kong et al. 2023; Cho et al. 2025), including cofetuzumab pelidotin (PF‐06647020/ABBV‐647), have been evaluated in lung cancer. Notably, a Phase 1b study of cofetuzumab pelidotin demonstrated preliminary antitumor activity (NCT04189614) despite adverse events such as alopecia and neutropenia (Cho et al. 2025). In addition, several investigational PTK7‐targeted ADCs are in clinical development, such as DAY301 (NCT06752681), KIVU‐107 (NCT07229313), LY4175408 (NCT07046923), and HWK‐007 (NCT07444814). Despite the ongoing clinical evaluation of multiple PTK7‐directed investigational agents (Mottard et al. 2025), no FDA‐approved therapies currently target PTK7, supporting its continued investigation as a potential target. To support its druggability, an analysis of RNAi gene effect scores from DepMap showed that PTK7 knockdown resulted in significantly more negative scores compared to random genes, suggesting PTK7 inhibition could effectively suppress cell growth.

Several limitations should be acknowledged. First, the lack of significant DEPs in the HNSCC data likely stems from mixed anatomical subsites (oral cavity *n* = 49, larynx *n* = 47, oropharynx *n* = 6, lip *n* = 4, hypopharynx *n* = 2). Future studies employing subsite‐stratified or adjusted models (Chung et al. 2004; Puram et al. 2017; Vidotto et al. 2018) would be essential to resolve this heterogeneity. Second, associations between clinical variables (age, sex, stage, BMI) and EV‐associated proteins remain exploratory due to limited sample size and lack of multivariable adjustment. For instance, while PKM abundance positively correlated with tumor stage in our LUAD cohort consistent with prior reports linking PKM upregulation to poorer outcomes (Guo et al. 2019; Long et al. 2020; Huang et al. 2017), future validation in larger cohorts with multivariate modeling is needed to confirm these associations. Third, Vesiclepedia‐based analysis is subject to publication bias; we therefore cross‐validated candidates using ExoCarta and HPA secretome databases (Table S6). Despite these limitations, our integrative framework successfully identified clinically relevant candidates across multiple cancer types, as exemplified by PTK7's ongoing clinical evaluation.

In conclusion, our study curated and integrated diverse cancer proteomics with EV catalog, providing a systematic framework for identifying and prioritizing candidate therapeutic targets. Through this integrated approach, we identified 26 RKs that are not only associated with EVs but also exhibit clinical significance in cancer. Our findings on EV‐associated RK targets and their associated drugs provide the fundamental knowledge that may inform the development of novel therapeutic approaches targeting these cancer proteins.

## Author Contributions

**Jina Kim**: conceptualization, methodology, formal analysis, writing – original draft, writing – review and editing, visualization, investigation. **Su Yeon Yeon**: conceptualization, methodology, writing – original draft, writing – review and editing, visualization. **Kyerim Choi**: formal analysis, writing – review and editing, investigation. **Hojung Kim**: formal analysis, writing – review and editing. **Hyoyoung Kim**: formal analysis, writing – review and editing, visualization. **Daehee Hwang**: writing – review and editing, project administration, investigation. **Sungyong You**: conceptualization, methodology, writing –original draft, writing – review and editing, supervision, project administration, investigation.

## Funding

This research was funded by National Institutes of Health R01CA277530 (H.R.T., Y.Z., V.G.A., J.D.Y., S.Y.), R01CA255727 (Y.Z., H.R.T., S.Y.), R01CA253651 (H.R.T., V.G.A., S.Y.), R01CA253651‐04S1 (Y.Z., H.R.T., S.Y.), R01CA246304 (H.R.T., V.G.A., S.Y.), P01CA278732 (S.Y.), and The Samuel Oschin Comprehensive Cancer Institute (SOCCI) at Cedars‐Sinai Medical Center through 2024 Program Project Grant (PPG) Team Science Award (S.Y.).

## Conflicts of Interest

The authors declare no conflicts of interest.

## Supporting information




**Supplementary Material**: jev270275‐sup‐0001‐SuppMat.xlsx

**Supplementary Material**: jev270275‐sup‐0002‐SuppMat.xlsx

**Supplementary Material**: jev270275‐sup‐0003‐SuppMat.docx

## Data Availability

This study is based on pan‐cancer proteome data from the Proteomic Data Commons of the National Cancer Institute (https://pdc.cancer.gov). EV quantity was sourced from Hoshino et al. (Hoshino et al. 2020) and Vesiclepedia data was used to indicate proteins commonly reported in EV studies (http://microvesicles.org) (Pathan et al. 2019), respectively. Plasma protein data was obtained from the Human Protein Atlas (Uhlén et al. 2019), and GESP scores were obtained from The Cancer Surfaceome Atlas (TCSA) (Hu et al. 2021). We obtained the drug sensitivity data and gene effect scores from DepMap (https://depmap.org) (Tsherniak et al. 2017, Mcfarland et al. 2018). The data curated by the present study are publicly available through Figshare (https://doi.org/10.6084/m9.figshare.24030126). All other data supporting the findings of the present study are available from the corresponding author on reasonable request.
