# Supplementary material for: Leveraging Quantitative Proteomics and Extracellular Vesicle Data to Uncover Druggable Receptor Kinases Across Cancers
Source: J Extracell Vesicles. 2026 Apr 14;15(4):e70275. doi: 10.1002/jev2.70275 (PMC13077551; doi:10.1002/jev2.70275)
Supplement: Supplementary file 3 — Supplementary Material: jev270275‐sup‐0003‐SuppMat.docx [file JEV2-15-e70275-s001.docx]

Supplementary Materials for

Leveraging Quantitative Proteomics and Extracellular Vesicle Data to Uncover Druggable Receptor Kinases Across Cancers

Jina Kim, Su Yeon Yeon, Kyerim Choi, Hojung Kim, HyoYoung Kim, Daehee Hwang, Sungyong You

Correspondence to: Sungyong.You@cshs.org

**This PDF file includes:**

Supplementary Fig. S1 to S13

Supplementary Tables S1 to S8

**Supplementary Fig. S1 The overview of processing of the protein data**


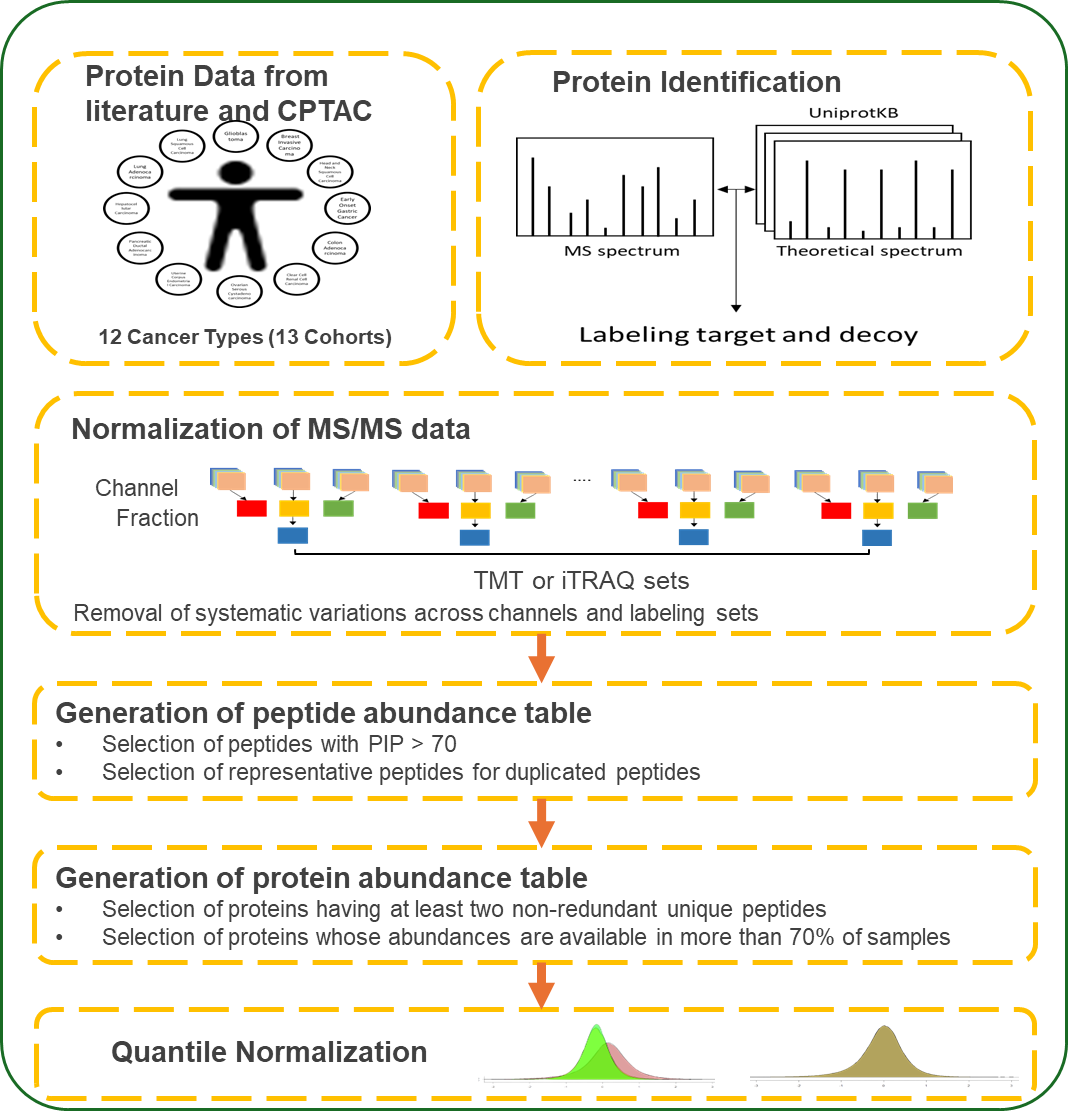


**Supplementary Fig. S2 The Cumulative Empirical Distribution of PIP values by Cohort**

EOGC


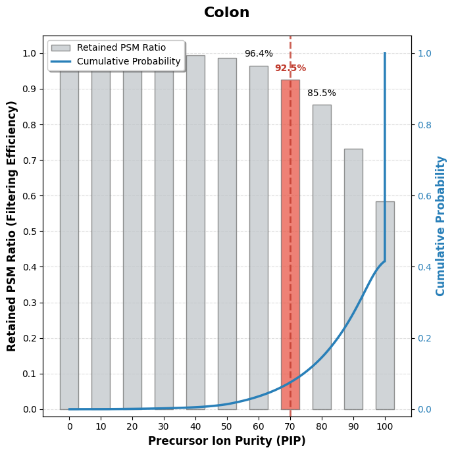

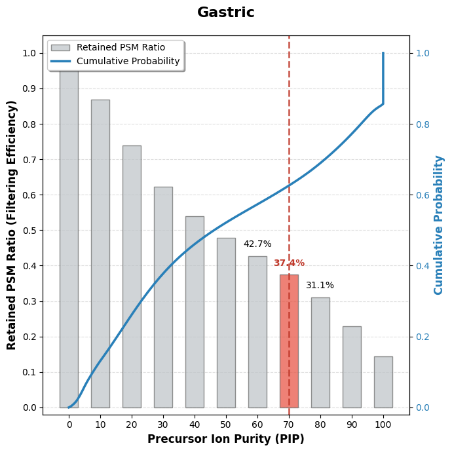

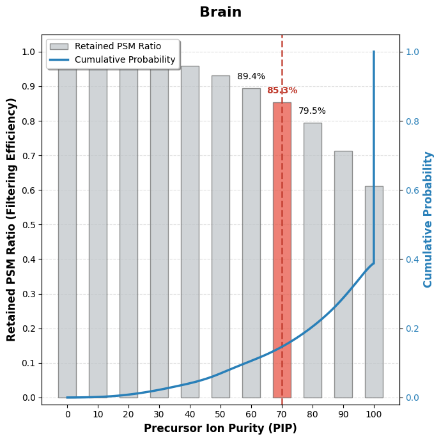

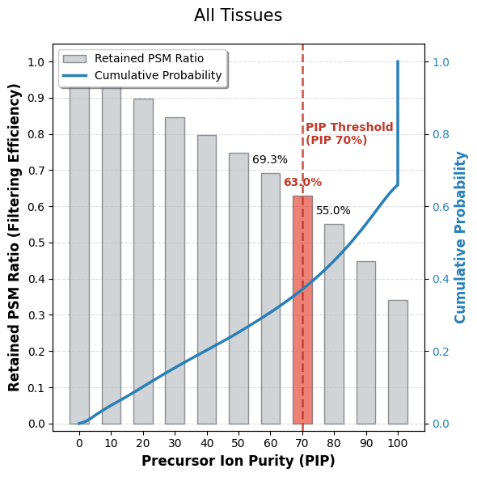

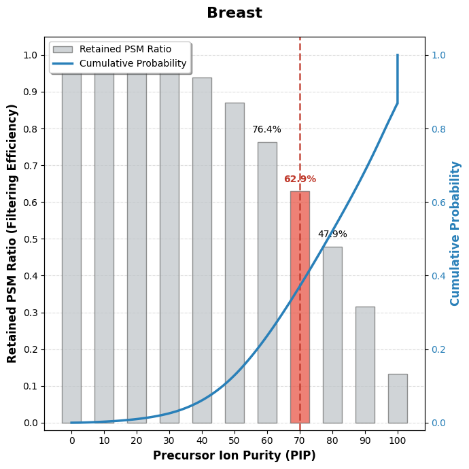


GBM

CRC

All Tissues

All Tissues

BRCA

HNSCC

LUSCC

PCJHU

HCC

LUAD


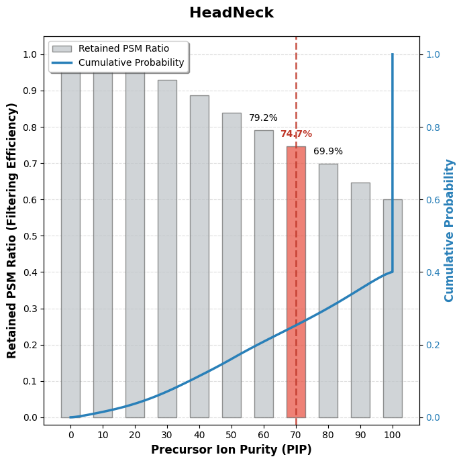

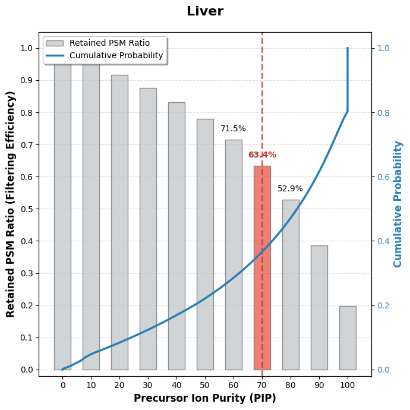

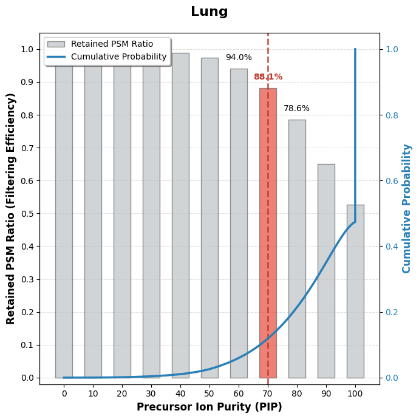

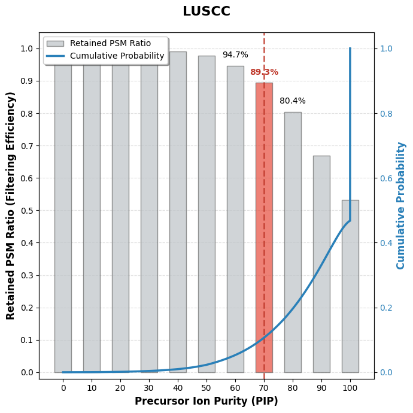

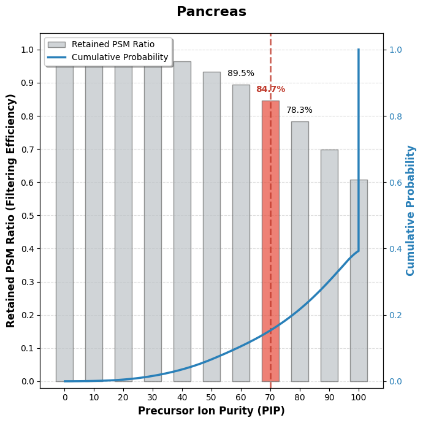


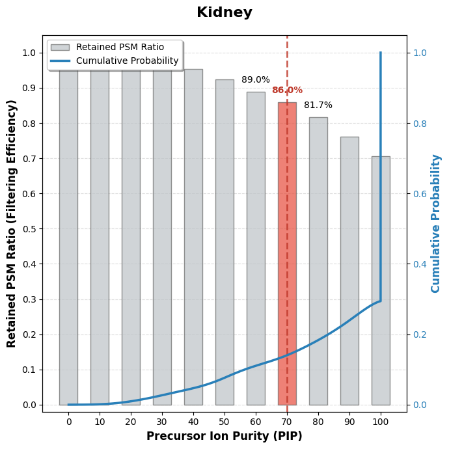


PCKU

RCC

UCEC

OV


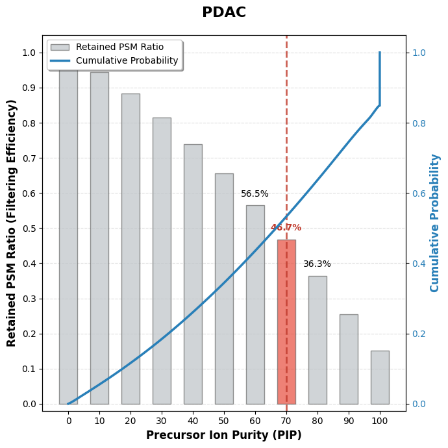

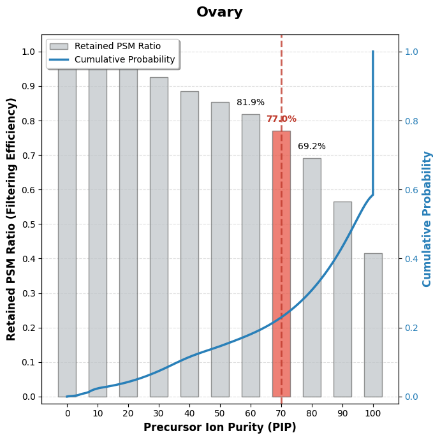

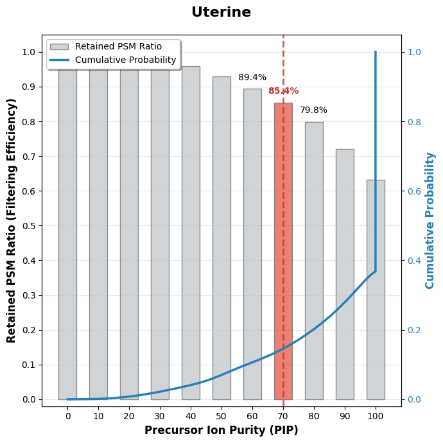


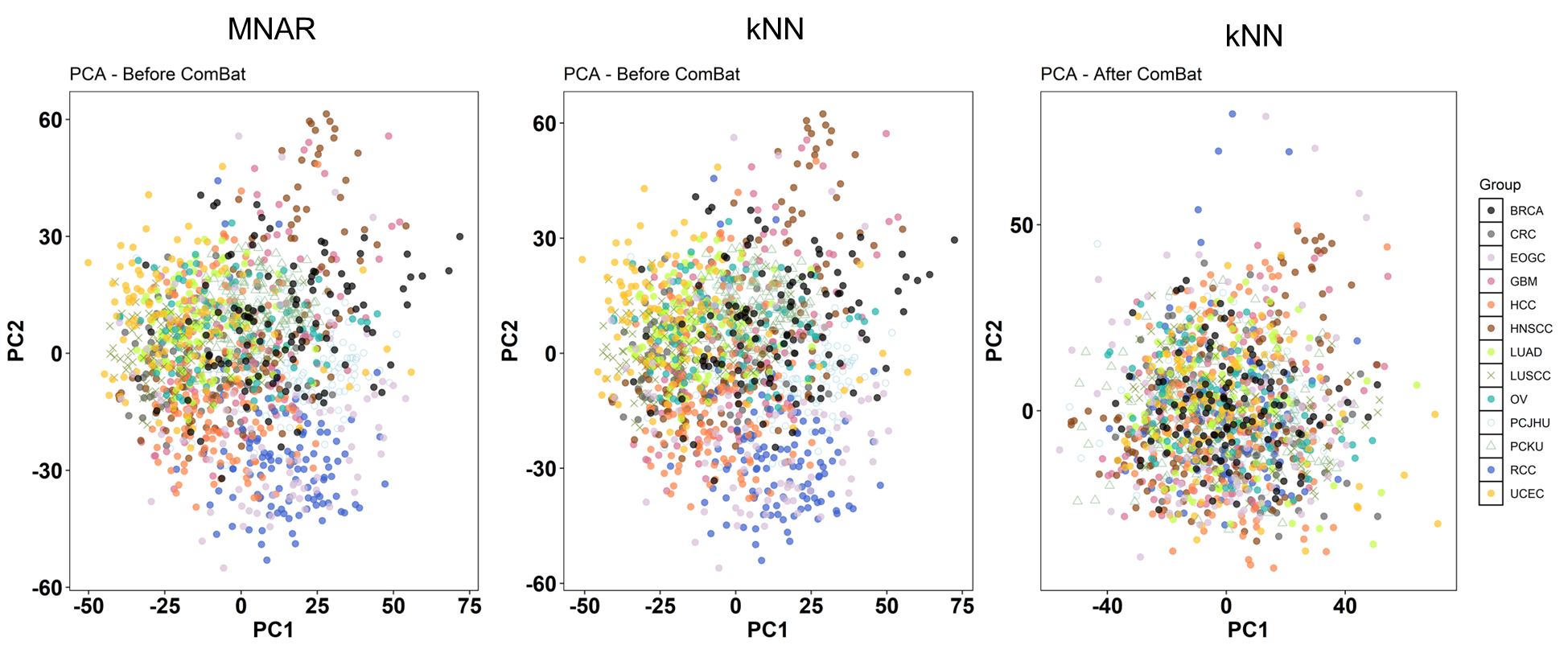


**Supplementary Fig. S3 PCA of the integrated imputed proteomics dataset before and after batch correction.**

PC1 versus PC2 is shown for all samples. Each point represents one sample, and colors/shapes indicate cohort/cancer type. Left: PCA before batch correction after MinProb low-value imputation (q = 0.01). Middle: PCA before batch correction after kNN imputation (k = 10). Right: PCA after ComBat batch correction (batch = cohort) applied to the kNN-imputed matrix, showing improved intermixing across cohorts.

**Supplementary Fig. S4 UMAP sensitivity analysis across combinations of n_neighbors (10/15/30) and min_dist (0.05/0.1/0.3) using the same input PCs (top 8) and random seed (42).**


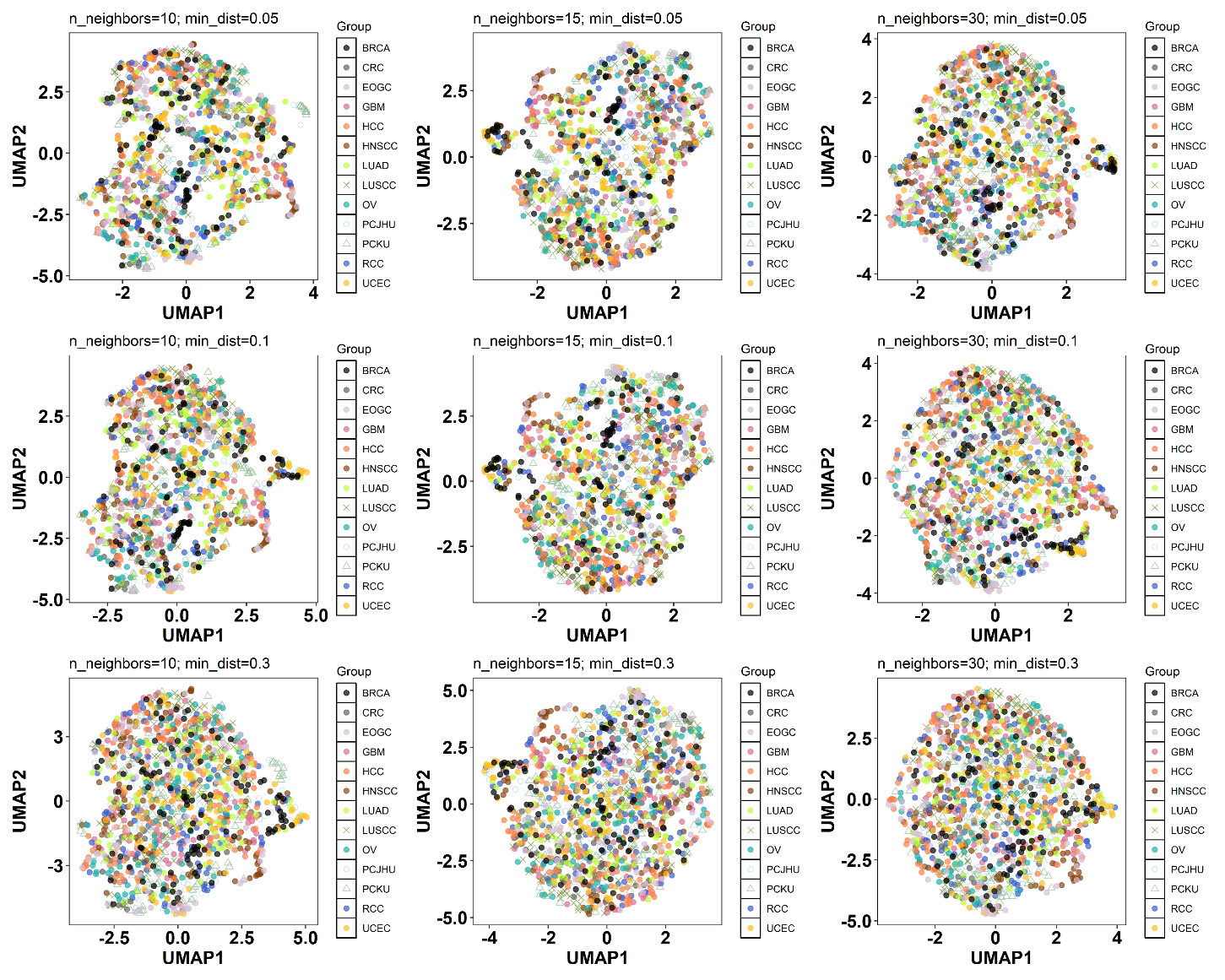


**Supplementary Fig. S5 Scree plots for selecting top PC (Principal Component). a. The scree plot of PC for total proteins b. The scree plot of PC for common EV proteins**


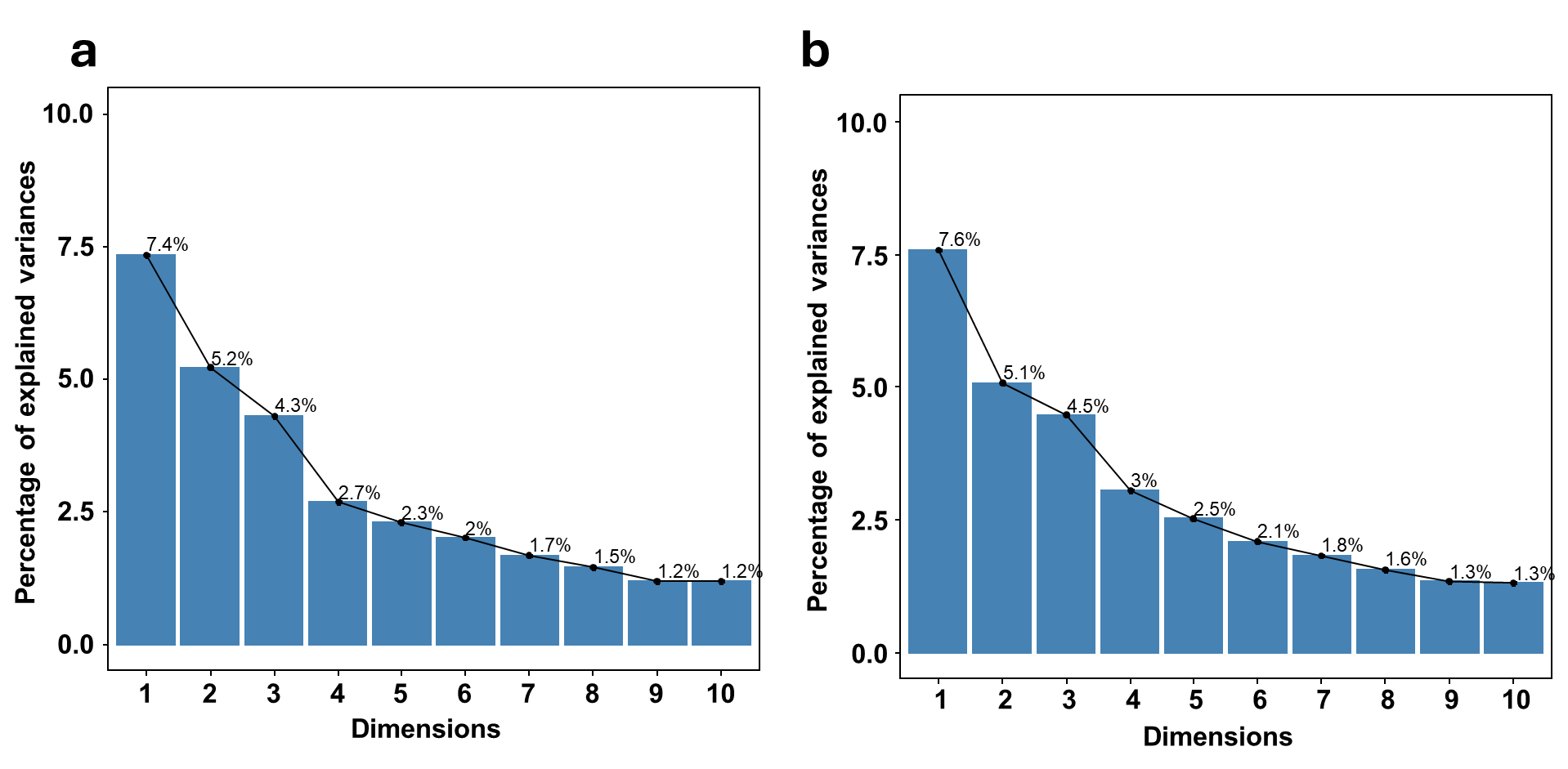


**Supplementary Fig. S6 The top 20 significant enriched GO cellular components (based on adjusted p-values) in each Ev protein(a) and non-Ev protein(b)**

**
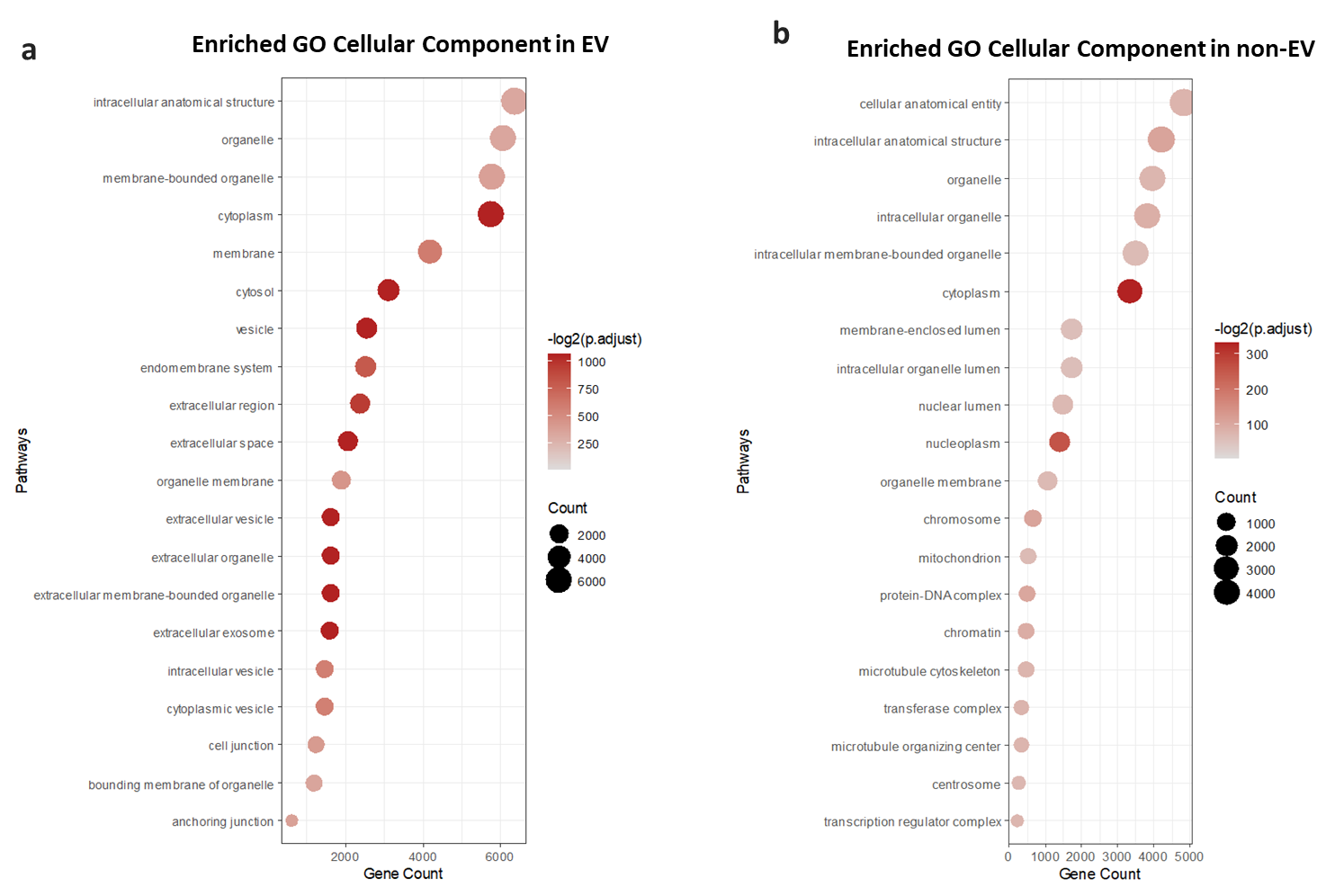
**

**Supplementary Fig. S7 Distribution of Wilcoxon p-values across cancer cohorts.** Histograms show the distribution of Wilcoxon test p-values calculated for each protein within individual cancer cohorts.

**
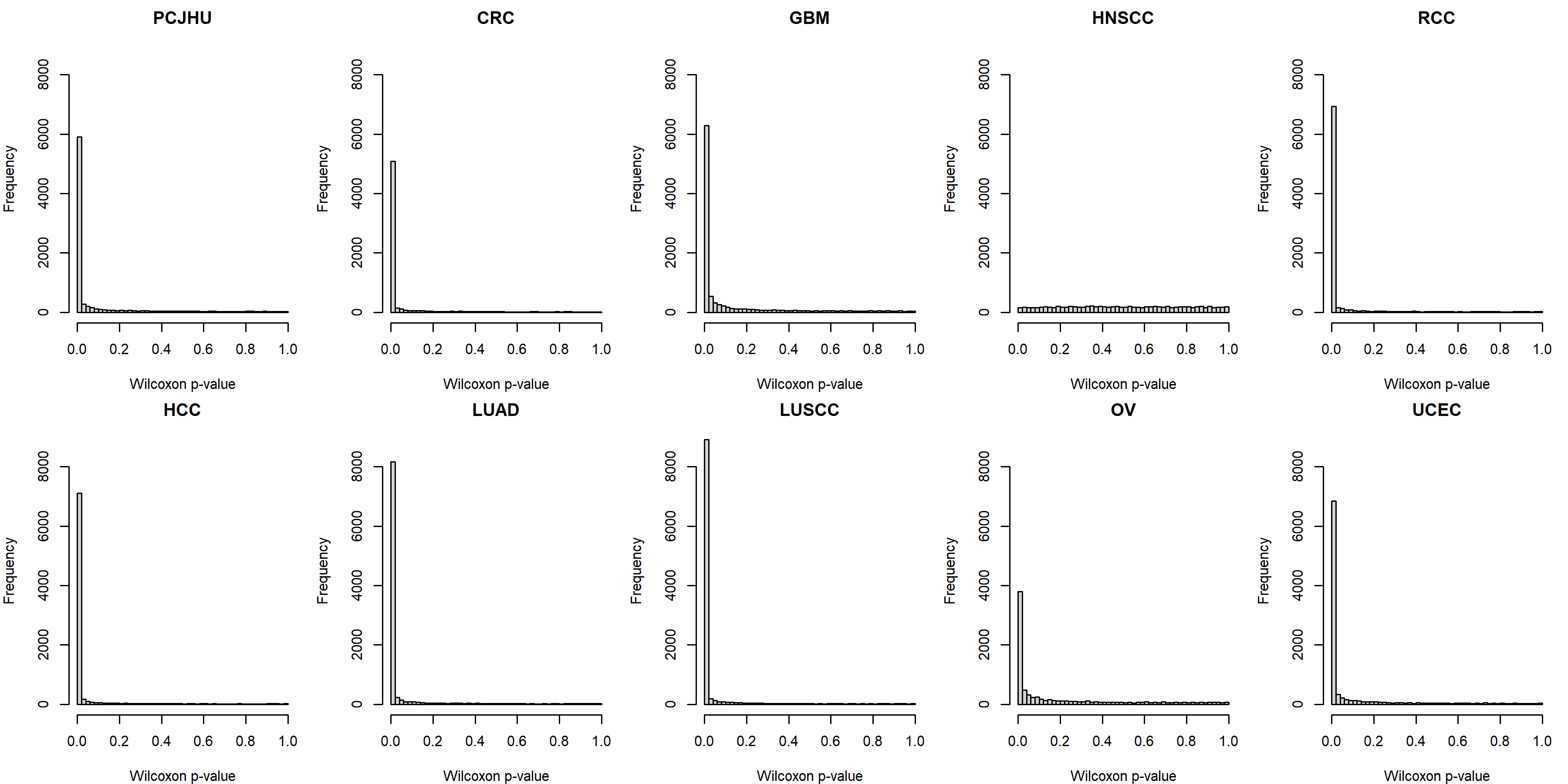
**

**Supplementary Fig. S8 Volcano plots of differential protein abundance across cancer cohorts.** Volcano plots depict differential protein abundance between comparison groups within each cancer cohort. The x-axis represents log2 fold change (log2FC), and the y-axis represents −log10 of the FDR-adjusted p-values derived from Wilcoxon tests. **
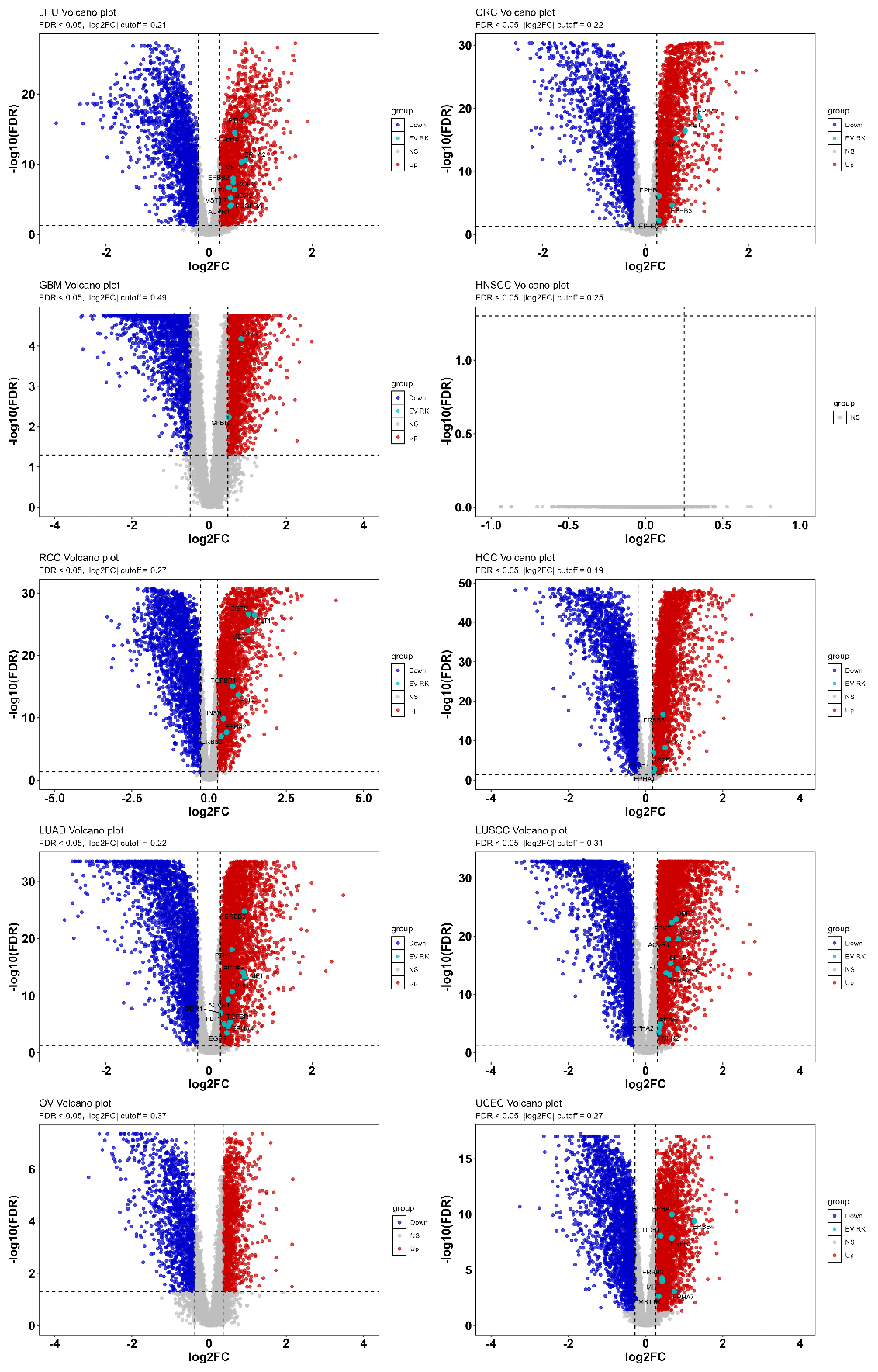
**

**Supplementary Fig. S9. Distribution of PTK7 abundance in HCC and determination of the optimal cutoff value. a. The distribution of PTK7 abundance in HCC and optimal cutoff value (0.3744).** This optimal cutoff value isolates a biologically plausible tail/peak rather than splitting the middle of a dense unimodal distribution. **b. Determination of optimal cutoff value and bootstrap validation.** Optimal cutoff value determination using the Youden index and its robustness assessed by bootstrap resampling (1,000 iterations). The optimal cutoff values across 1,000 iterations were highly centered (0.25-0.5) around the selected cutoff (bottom-left), suggesting that our dichotomization is robust and minimizes the risk of Type I errors.

**b**


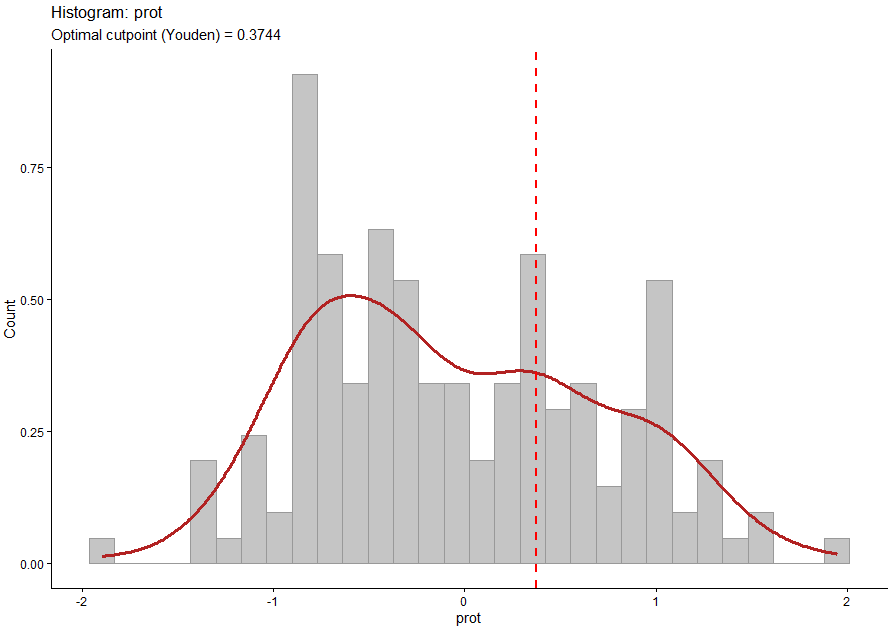

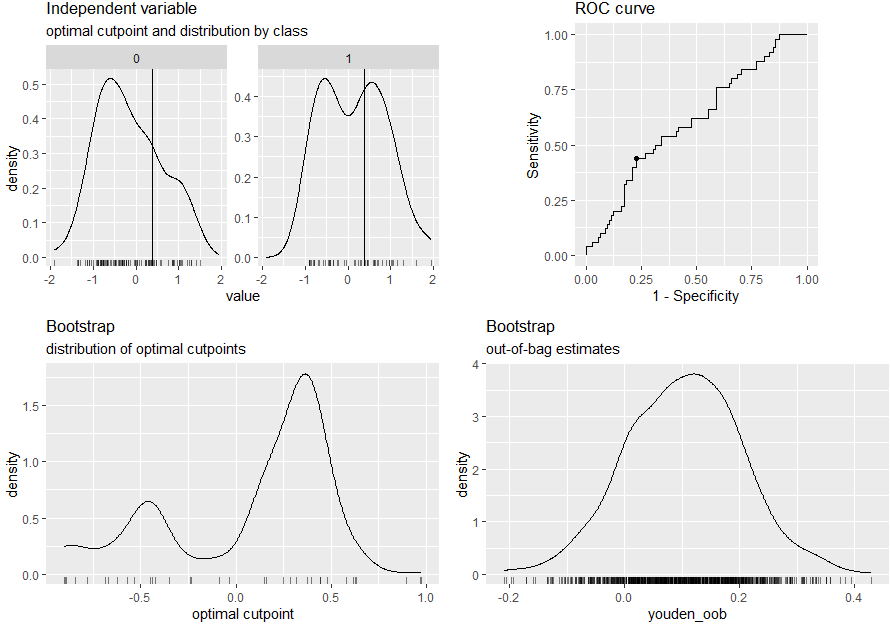


**a**

**Supplementary Fig. S10 The proportions by protein class of specific proteins detected in only one cancer cohort**


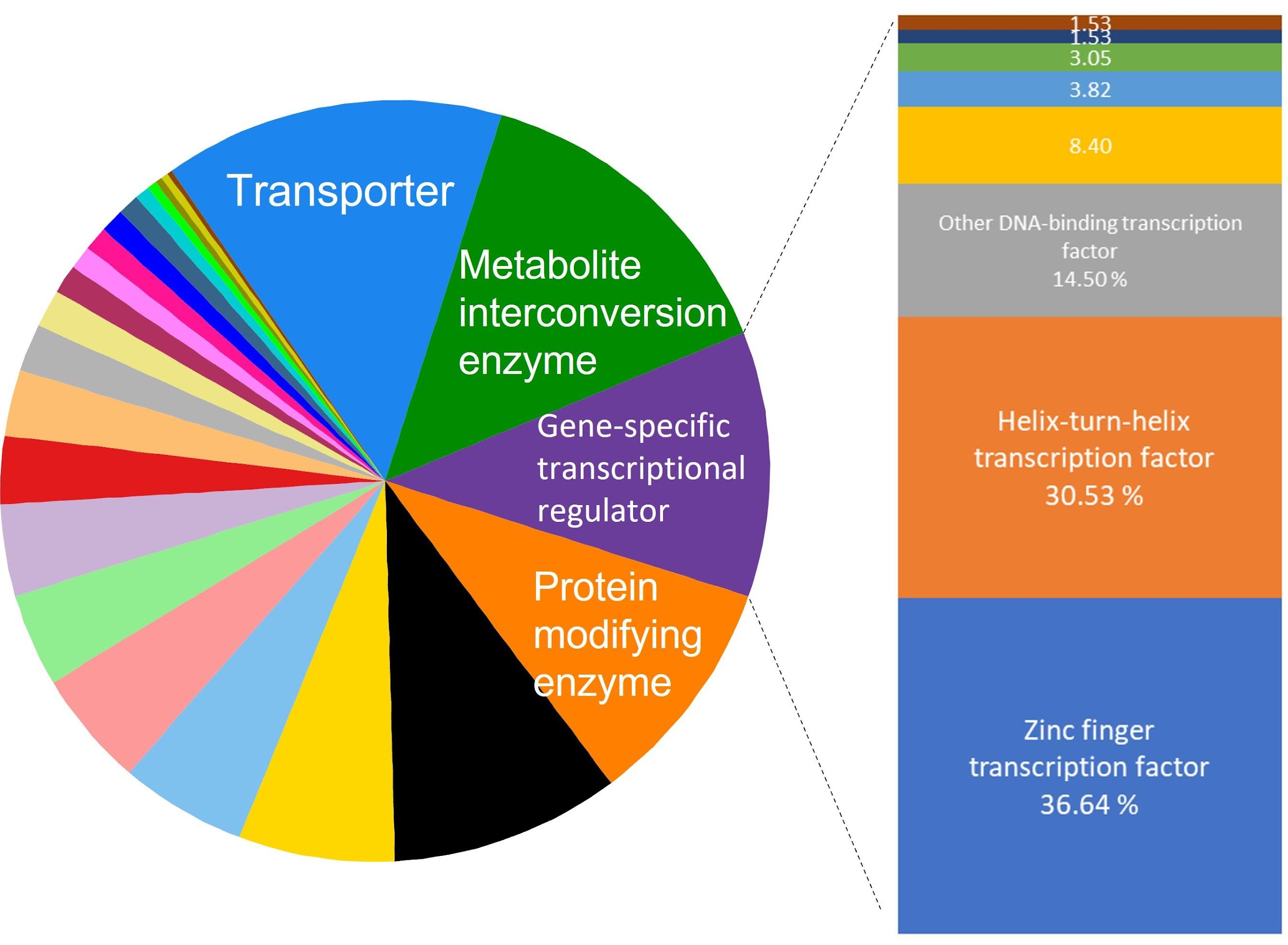


**Supplementary Fig. S11 Kaplan-Meier survival plots of low versus high protein expression groups for genes of HR CI >1 by cancer types**

**
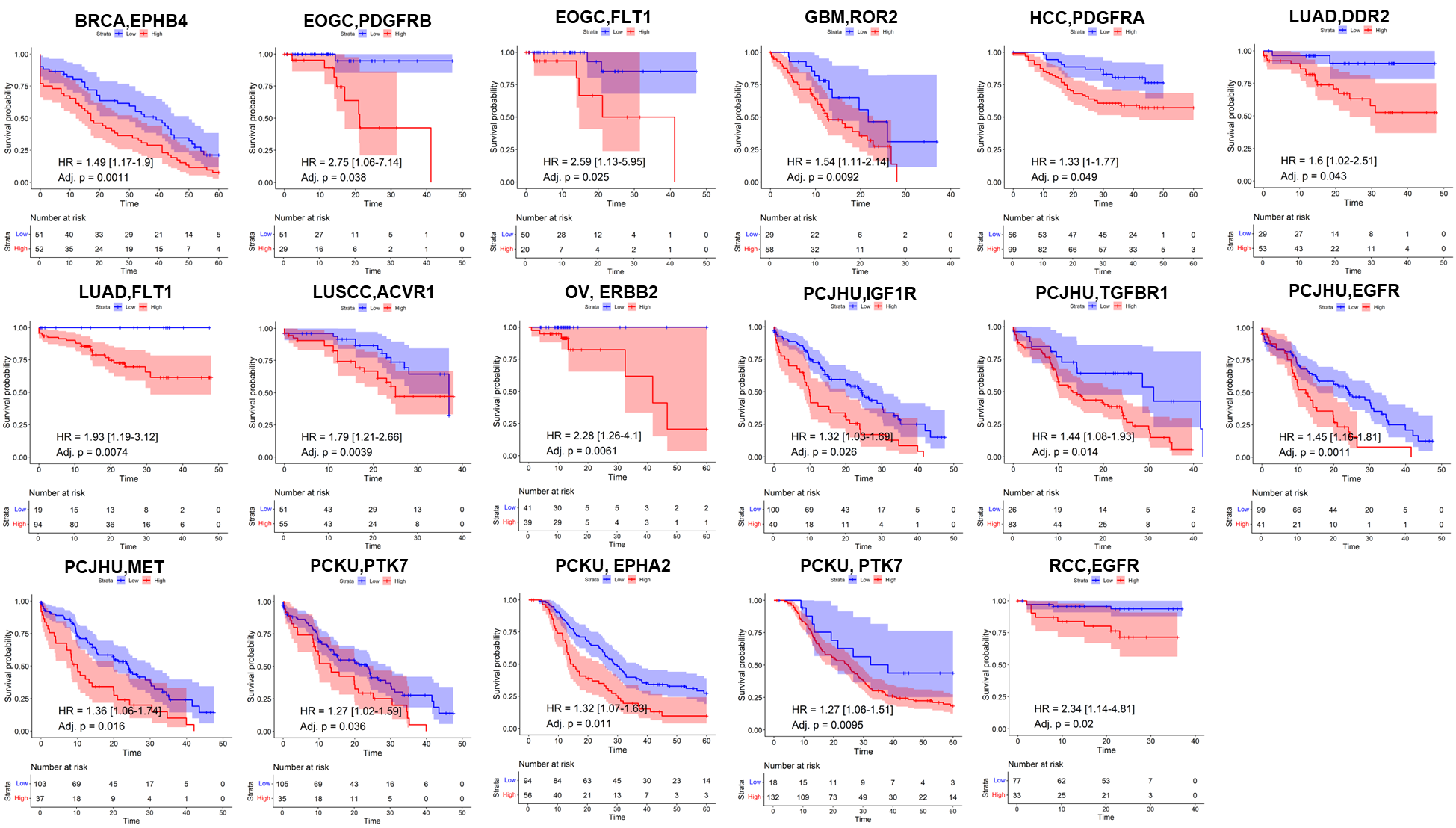
**

**Supplementary Fig. S12 The median LFC(log2 fold-change viability) values by cancer types of FDA-approved drugs in our network from DepMap data**

**
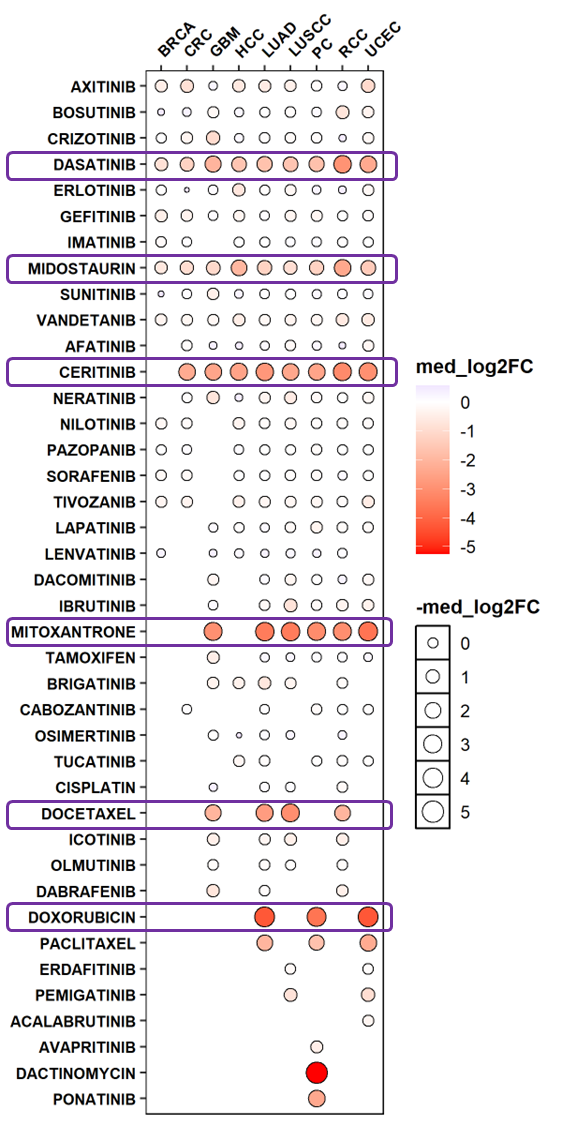
**

**Supplementary Fig. S13 Comparison of RNAi gene effect scores between PTK7 and randomly selected genes**

**
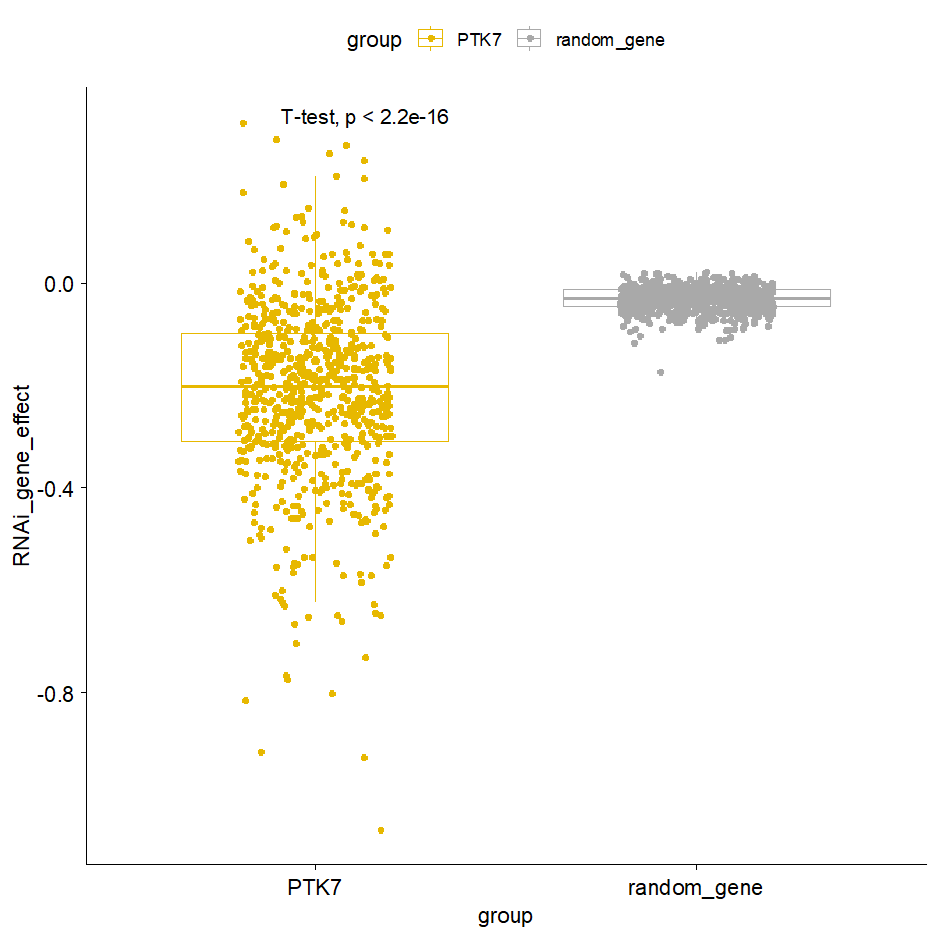
**

**Supplementary Table S1. The count of proteins, genes after data grouping and filtering process, and samples by cancer types**

| Cancer type | Number of proteins  (UniProt IDs) | Number of genes for proteins  (Entrez IDs) | Total  sample | Tumor  sample | Normal  sample |
| --- | --- | --- | --- | --- | --- |
| CRC | 6,436 | 6,429 | 197 | 97 | 100 |
| GBM | 10,305 | 10,285 | 110 | 100 | 10 |
| LUAD | 9,932 | 9,917 | 215 | 113 | 102 |
| OV | 8,491 | 8,474 | 103 | 84 | 19 |
| UCEC | 9,611 | 9,592 | 149 | 100 | 49 |
| EOGC | 7,542 | 7,538 | 80 | 80 | 0 |
| RCC | 8,452 | 8,441 | 207 | 110 | 97 |
| HCC | 8,404 | 8,395 | 320 | 160 | 160 |
| HNSCC | 8,865 | 8,854 | 180 | 115 | 65 |
| BRCA | 8,045 | 8,033 | 108 | 108 | 0 |
| LUSCC | 10,531 | 10,510 | 212 | 110 | 102 |
| PCJHU | 8,469 | 8,455 | 238 | 145 | 93 |
| PCKU | 8,468 | 8,448 | 153 | 153 | 0 |

**Supplementary Table S2. Summary of clinical characteristics across 13 datasets of 12 different cancer types**

| Cancer Type | No. of Cases | Age (Mean± SD) | Sex | | Race | | | | | | BMI (Mean± SD) | Stage | |
| --- | --- | --- | --- | --- | --- | --- | --- | --- | --- | --- | --- | --- | --- |
|  |  |  | M | F | White | Black | Asian | Hispanic | AmericanIndian | Unknown |  | Stage 1-2 | Stage 3-4 |
| LUAD | 111 | 63± 9.6 | 73 | 38 | 41 | 1 | 64 | 2 | 0 | 3 | 24.4±6.29 | 89 | 22 |
| LUSCC | 110 | 66± 8.1 | 88 | 22 | 83 | 1 | 24 | 0 | 0 | 2 | 26.4±4.89 | 85 | 22 |
| GBM | 99 | 58± 12.5 | 55 | 44 | 66 | 0 | 31 | 2 | 0 | 0 | 26.2±4.52 | NA | |
| HNSCC | 108 | 61± 8.8 | 94 | 14 | 97 | 1 | 7 | 1 | 0 | 2 | 24.9±5.19 | 32 | 76 |
| PCJHU | 140 | 61± 12.4 | 74 | 66 | 108 | 2 | 24 | 0 | 0 | 6 | 25.2±4.92 | 83 | 42 |
| PCKU | 196 | 65± 10.1 | 112 | 84 | 0 | 0 | 196 | 0 | 0 | 0 | NA | 122 | 28 |
| RCC | 110 | 60± 12.2 | 80 | 30 | 103 | 1 | 5 | 0 | 0 | 1 | 31.4±8.79 | 65 | 45 |
| HCC | 160 | 53± 10.9 | 128 | 32 | 0 | 0 | 160 | 0 | 0 | 0 | NA | 104 | 53 |
| EOGC | 80 | 37± 5.6 | 36 | 44 | 0 | 0 | 80 | 0 | 0 | 0 | NA | 31 | 49 |
| CRC | 97 | 65± 11.5 | 41 | 56 | 71 | 7 | 16 | 0 | 1 | 2 | 28.6±8.53 | 49 | 48 |
| OV | 111 | 59± 9.8 | 0 | 111 | 95 | 3 | 9 | 0 | 2 | 2 | NA | 1 | 77 |
| BRCA | 105 | 59± 13.1 | 2 | 103 | NA | | | | | | NA | 76 | 29 |
| UCEC | 100 | 64± 10.1 | 0 | 100 | 95 | 3 | 1 | 0 | 0 | 1 | 35±10.3 | 82 | 18 |

**Supplementary Table S3. The protein classes of common cancer genes and one cancer specific genes by Panther DB**

| The classification of common cancer genes | | | The classification of one cancer specific genes | | |
| --- | --- | --- | --- | --- | --- |
| **Protein class** | **The number of Genes** | **Percents (%)** | **Protein class** | **The number of Genes** | **Percents (%)** |
| metabolite interconversion enzyme (PC00262) | 807 | 19.90% | transporter (PC00227) | 166 | 14.30% |
| protein modifying enzyme (PC00260) | 523 | 12.90% | metabolite interconversion enzyme (PC00262) | 164 | 14.20% |
| RNA metabolism protein (PC00031) | 473 | 11.70% | gene-specific transcriptional regulator (PC00264) | 131 | 11.30% |
| protein-binding activity modulator (PC00095) | 291 | 7.20% | protein modifying enzyme (PC00260) | 112 | 9.70% |
| translational protein (PC00263) | 273 | 6.70% | transmembrane signal receptor (PC00197) | 111 | 9.60% |
| cytoskeletal protein (PC00085) | 253 | 6.30% | scaffold/adaptor protein (PC00226) | 76 | 6.60% |
| scaffold/adaptor protein (PC00226) | 225 | 5.60% | protein-binding activity modulator (PC00095) | 62 | 5.40% |
| membrane traffic protein (PC00150) | 212 | 5.20% | cell adhesion molecule (PC00069) | 58 | 5.00% |
| transporter (PC00227) | 198 | 4.90% | intercellular signal molecule (PC00207) | 46 | 4.00% |
| chaperone (PC00072) | 136 | 3.40% | cytoskeletal protein (PC00085) | 45 | 3.90% |
| gene-specific transcriptional regulator (PC00264) | 114 | 2.80% | membrane traffic protein (PC00150) | 34 | 2.90% |
| chromatin/chromatin-binding, or -regulatory protein (PC00077) | 104 | 2.60% | defense/immunity protein (PC00090) | 32 | 2.80% |
| defense/immunity protein (PC00090) | 87 | 2.20% | RNA metabolism protein (PC00031) | 23 | 2.00% |
| DNA metabolism protein (PC00009) | 81 | 2.00% | structural protein (PC00211) | 18 | 1.60% |
| transfer/carrier protein (PC00219) | 55 | 1.40% | extracellular matrix protein (PC00102) | 14 | 1.20% |
| extracellular matrix protein (PC00102) | 53 | 1.30% | DNA metabolism protein (PC00009) | 12 | 1.00% |
| cell adhesion molecule (PC00069) | 54 | 1.30% | chromatin/chromatin-binding, or -regulatory protein (PC00077) | 11 | 1.00% |
| calcium-binding protein (PC00060) | 37 | 0.90% | transfer/carrier protein (PC00219) | 11 | 1.00% |
| transmembrane signal receptor (PC00197) | 28 | 0.70% | calcium-binding protein (PC00060) | 10 | 0.90% |
| intercellular signal molecule (PC00207) | 18 | 0.40% | cell junction protein (PC00070) | 7 | 0.60% |
| structural protein (PC00211) | 12 | 0.30% | viral or transposable element protein (PC00237) | 5 | 0.40% |
| cell junction protein (PC00070) | 7 | 0.20% | translational protein (PC00263) | 3 | 0.30% |
| viral or transposable element protein (PC00237) | 3 | 0.10% | chaperone (PC00072) | 4 | 0.30% |
| storage protein (PC00210) | 2 | 0.00% | storage protein (PC00210) | 2 | 0.20% |
| Percents (%) = (the number of genes by class / classified total genes)*100, | | | | | |

**Supplementary Table S4. The protein classes of GBM specific genes by Panther DB**

| **Protein class** | **The number of Genes** | **Percents (%)** |
| --- | --- | --- |
| transporter (PC00227)* | 88 | 16.60% |
| transmembrane signal receptor (PC00197) | 75 | 14.20% |
| protein modifying enzyme (PC00260) | 50 | 9.50% |
| metabolite interconversion enzyme (PC00262) | 49 | 9.30% |
| scaffold/adaptor protein (PC00226) | 48 | 9.10% |
| cell adhesion molecule (PC00069) | 44 | 8.30% |
| protein-binding activity modulator (PC00095) | 36 | 6.80% |
| gene-specific transcriptional regulator (PC00264) | 27 | 5.10% |
| membrane traffic protein (PC00150) | 25 | 4.70% |
| intercellular signal molecule (PC00207) | 15 | 2.80% |
| cytoskeletal protein (PC00085) | 11 | 2.10% |
| defense/immunity protein (PC00090) | 10 | 1.90% |
| extracellular matrix protein (PC00102) | 9 | 1.70% |
| RNA metabolism protein (PC00031) | 9 | 1.70% |
| calcium-binding protein (PC00060) | 9 | 1.70% |
| structural protein (PC00211) | 7 | 1.30% |
| transfer/carrier protein (PC00219) | 6 | 1.10% |
| cell junction protein (PC00070) | 4 | 0.80% |
| translational protein (PC00263) | 3 | 0.60% |
| chaperone (PC00072) | 2 | 0.40% |
| viral or transposable element protein (PC00237) | 1 | 0.20% |
| chromatin/chromatin-binding, or -regulatory protein (PC00077) | 1 | 0.20% |
| Percents (%) = (the number of genes by class / classified total genes)*100, | | |
| *Transporter group = Ion channel (66.70 %) + secondary carrier transporter (18.20 %) + primary active transporter (14.30 %) | | |

**Supplementary Table S5. The significance score of 26 Receptor Kinases by cancer groups**

|  |  | **DEP significance** | | | | | | | | | | | **HR significance** | | | | **DEP + HR significance** | |
| --- | --- | --- | --- | --- | --- | --- | --- | --- | --- | --- | --- | --- | --- | --- | --- | --- | --- | --- |
| **Gene** | **UNIPROT** | **LUSCC** | **LUAD** | **HCC** | **RCC** | **PCJHU** | **GBM** | **CRC** | **UCEC** | **OV** | **HNSCC** | **BRCA** | | **PCKU** | **GA** | **Total** | |  |
| PTK7 | Q13308 | 1 | 1 | 1 | 0 | 1 | 1 | 1 | 0 | 0 | 0 | 0 | | 1 | 0 | 7 | |  |
| MET | P08581 | 0 | 1 | 0 | 1 | 1 | 0 | 1 | 1 | 0 | 0 | 0 | | 1 | 0 | 6 | |  |
| FLT1 | P17948 | 1 | 1 | 1 | 1 | 1 | 0 | 0 | 0 | 0 | 0 | 0 | | 0 | 1 | 6 | |  |
| EPHA2 | P29317 | 1 | 0 | 0 | 1 | 1 | 0 | 1 | 0 | 0 | 0 | 0 | | 1 | 0 | 5 | |  |
| EGFR | P00533 | 1 | 1 | 0 | 1 | 0 | 1 | 0 | 0 | 0 | 0 | 0 | | 0 | 0 | 4 | |  |
| EPHB3 | P54753 | 1 | 1 | 0 | 0 | 0 | 0 | 1 | 1 | 0 | 0 | 0 | | 0 | 0 | 4 | |  |
| ACVR1 | Q04771 | 1 | 1 | 1 | 0 | 1 | 0 | 0 | 0 | 0 | 0 | 0 | | 0 | 0 | 4 | |  |
| ERBB2 | P04626 | 0 | 1 | 0 | 0 | 1 | 0 | 0 | 1 | 0 | 0 | 0 | | 0 | 0 | 3 | |  |
| DDR1 | Q08345 | 1 | 1 | 0 | 0 | 0 | 0 | 0 | 1 | 0 | 0 | 0 | | 0 | 0 | 3 | |  |
| EPHB2 | P29323 | 1 | 1 | 0 | 0 | 0 | 0 | 1 | 0 | 0 | 0 | 0 | | 0 | 0 | 3 | |  |
| TGFBR1 | P36897 | 0 | 1 | 0 | 1 | 0 | 1 | 0 | 0 | 0 | 0 | 0 | | 0 | 0 | 3 | |  |
| ERBB3 | P21860 | 0 | 0 | 1 | 1 | 0 | 0 | 0 | 1 | 0 | 0 | 0 | | 0 | 0 | 3 | |  |
| EPHA1 | P21709 | 1 | 0 | 1 | 0 | 0 | 0 | 0 | 1 | 0 | 0 | 0 | | 0 | 0 | 3 | |  |
| EPHB4 | P54760 | 0 | 0 | 0 | 0 | 0 | 0 | 1 | 0 | 0 | 0 | 1 | | 0 | 0 | 2 | |  |
| MST1R | Q04912 | 0 | 0 | 0 | 0 | 1 | 0 | 0 | 1 | 0 | 0 | 0 | | 0 | 0 | 2 | |  |
| INSR | P06213 | 0 | 0 | 1 | 1 | 0 | 0 | 0 | 0 | 0 | 0 | 0 | | 0 | 0 | 2 | |  |
| EPHA4 | P54764 | 1 | 1 | 0 | 0 | 0 | 0 | 0 | 0 | 0 | 0 | 0 | | 0 | 0 | 2 | |  |
| FGFR2 | P21802 | 1 | 0 | 0 | 0 | 0 | 0 | 0 | 1 | 0 | 0 | 0 | | 0 | 0 | 2 | |  |
| PDGFRB | P09619 | 0 | 0 | 0 | 0 | 1 | 0 | 0 | 0 | 0 | 0 | 0 | | 0 | 1 | 2 | |  |
| ROR2 | Q01974 | 0 | 0 | 0 | 0 | 1 | 0 | 0 | 0 | 0 | 0 | 0 | | 0 | 0 | 1 | |  |
| DDR2 | Q16832 | 0 | 0 | 0 | 0 | 1 | 0 | 0 | 0 | 0 | 0 | 0 | | 0 | 0 | 1 | |  |
| IGF1R | P08069 | 1 | 0 | 0 | 0 | 0 | 0 | 0 | 0 | 0 | 0 | 0 | | 0 | 0 | 1 | |  |
| PDGFRA | P16234 | 0 | 0 | 0 | 0 | 1 | 0 | 0 | 0 | 0 | 0 | 0 | | 0 | 0 | 1 | |  |
| FLT4 | P35916 | 0 | 0 | 0 | 1 | 0 | 0 | 0 | 0 | 0 | 0 | 0 | | 0 | 0 | 1 | |  |
| ERBB4 | Q15303 | 0 | 0 | 0 | 0 | 0 | 0 | 0 | 1 | 0 | 0 | 0 | | 0 | 0 | 1 | |  |
| EPHA7 | Q15375 | 0 | 0 | 0 | 0 | 0 | 0 | 0 | 1 | 0 | 0 | 0 | | 0 | 0 | 1 | |  |

|  |  | **ExoCarta / HPA secretome: detected** | **Bausch-Fluck D. et al (2015, 2018)** | | |
| --- | --- | --- | --- | --- | --- |
| **Gene** | **UNIPROT** | **ExoCarta / HPA secretome: detected (Y/N)** | **TM domains**  **(Counts)** | **Signalpeptide (0/1)** | **Topology source** |
| PTK7 | Q13308 | **Y (ExoCarta / HPA secretome)** | **1** | **1** | **uniprot - CSPA confirmed** |
| MET | P08581 | **Y (ExoCarta / HPA secretome)** | **1** | **1** | **uniprot - CSPA confirmed** |
| FLT1 | P17948 | **Y (ExoCarta / HPA secretome)** | **1** | **1** | **uniprot - CSPA confirmed** |
| EPHA2 | P29317 | **Y (ExoCarta)** | **1** | **1** | **uniprot - CSPA confirmed** |
| EGFR | P00533 | **Y (ExoCarta / HPA secretome)** | **1** | **1** | **uniprot - CSPA confirmed** |
| EPHB3 | P54753 | **Y (ExoCarta)** | **1** | **1** | **uniprot - CSPA confirmed** |
| ACVR1 | Q04771 | **Y (ExoCarta)** | **1** | **1** | **uniprot - CSPA confirmed** |
| ERBB2 | P04626 | **Y (ExoCarta)** | **1** | **1** | **uniprot - CSPA confirmed** |
| DDR1 | Q08345 | **Y (ExoCarta HPA secretome)** | **1** | **1** | **uniprot** |
| EPHB2 | P29323 | **Y (ExoCarta / HPA secretome)** | **1** | **1** | **uniprot - CSPA confirmed** |
| TGFBR1 | P36897 | **Y (ExoCarta / HPA secretome)** | **1** | **1** | **uniprot** |
| ERBB3 | P21860 | **Y (ExoCarta / HPA secretome)** | **1** | **1** | **uniprot - CSPA confirmed** |
| EPHA1 | P21709 | **Y (ExoCarta)** | **1** | **1** | **uniprot - CSPA confirmed** |
| EPHB4 | P54760 | **Y (ExoCarta)** | **1** | **1** | **uniprot - CSPA confirmed** |
| MST1R | Q04912 | **Y (ExoCarta)** | **1** | **1** | **uniprot - CSPA confirmed** |
| INSR | P06213 | **Y (ExoCarta)** | **1** | **1** | **uniprot - CSPA confirmed** |
| EPHA4 | P54764 | **Y (ExoCarta)** | **1** | **1** | **uniprot - CSPA confirmed** |
| FGFR2 | P21802 | **Y (ExoCarta / HPA secretome)** | **1** | **1** | **uniprot - CSPA confirmed** |
| ROR2 | Q01974 | **Y (ExoCarta)** | **1** | **1** | **uniprot - CSPA confirmed** |
| DDR2 | Q16832 | **Y (ExoCarta)** | **1** | **1** | **uniprot - CSPA confirmed** |
| IGF1R | P08069 | **Y (ExoCarta)** | **1** | **1** | **uniprot - CSPA confirmed** |
| PDGFRB | P09619 | **Y (ExoCarta)** | **1** | **1** | **uniprot - CSPA confirmed** |
| PDGFRA | P16234 | **Y (ExoCarta / HPA secretome)** | **1** | **1** | **uniprot - CSPA confirmed** |
| FLT4 | P35916 | **Y (HPA secretome)** | **1** | **1** | **uniprot - CSPA confirmed** |
| ERBB4 | Q15303 | **Y (ExoCarta)** | **1** | **1** | **uniprot** |
| EPHA7 | Q15375 | **Y (ExoCarta / HPA secretome)** | **1** | **1** | **uniprot** |

**Supplementary Table S6. EV/secretome-supported receptor kinases and membrane topology features of 26 Receptor Kinase.**

**Supplementary Table S7. The association scores and literature scores of receptor kinase candidates in the associated each cancer.**

| **Disease** | **RK** | **Association Score(globalScore)** | **Literature Score(europepmc)** | **Novelty Score** |
| --- | --- | --- | --- | --- |
| brain cancer | EGFR | 0.5089 | 0.4268 | 0.039 |
| brain cancer | TGFBR1 | 0.0157 | 0.1292 | 0.866 |
| breast cancer | EPHB4 | 0.1309 | 0.6901 | 0.583 |
| breast cancer | FLT1 | 0.4003 | 0.9201 | 0.403 |
| clear cell renal carcinoma | EGFR | 0.3013 | 0.1430 | 0.297 |
| clear cell renal carcinoma | EPHA1 | 0.0762 | 0.0740 | 0.649 |
| clear cell renal carcinoma | EPHA2 | 0.0784 | 0.1488 | 0.593 |
| clear cell renal carcinoma | ERBB3 | 0.2959 | 0.0152 | 0.315 |
| clear cell renal carcinoma | FLT1 | 0.4009 | 0.1124 | 0.186 |
| clear cell renal carcinoma | FLT4 | 0.4797 | 0.0350 | 0 |
| clear cell renal carcinoma | INSR | 0.3473 | 0.0733 | 0.204 |
| clear cell renal carcinoma | MET | 0.3301 | 0.1071 | 0.241 |
| colon carcinoma | EPHA2 | 0.0790 | 0.1682 | 0.612 |
| colon carcinoma | EPHB2 | 0.0409 | 0.3362 | 0.667 |
| colon carcinoma | EPHB3 | 0.0474 | 0.3902 | 0.649 |
| colon carcinoma | EPHB4 | 0.0086 | 0.0707 | 0.815 |
| colon carcinoma | MET | 0.3939 | 0.2791 | 0.297 |
| colon carcinoma | PTK7 | 0.0209 | 0.1723 | 0.704 |
| hepatocellular carcinoma | ACVR1 | 0.3498 | 0.1103 | 0.377 |
| hepatocellular carcinoma | EPHA1 | 0.1153 | 0.7803 | 0.537 |
| hepatocellular carcinoma | ERBB3 | 0.3808 | 0.9060 | 0.305 |
| hepatocellular carcinoma | FLT1 | 0.4374 | 0.4705 | 0.218 |
| hepatocellular carcinoma | INSR | 0.0889 | 0.4927 | 0.638 |
| hepatocellular carcinoma | PTK7 | 0.0935 | 0.7691 | 0.609 |
| lung adenocarcinoma | ACVR1 | 0.3255 | 0.0684 | 0.323 |
| lung adenocarcinoma | DDR1 | 0.0437 | 0.3590 | 0.63 |
| lung adenocarcinoma | EGFR | 0.8293 | 0.9947 | 0 |
| lung adenocarcinoma | EPHA4 | 0.0115 | 0.0947 | 0.871 |
| lung adenocarcinoma | EPHB2 | 0.0936 | 0.7700 | 0.533 |
| lung adenocarcinoma | EPHB3 | 0.0071 | 0.0380 | 0.968 |
| lung adenocarcinoma | ERBB2 | 0.7568 | 0.8838 | 0.033 |
| lung adenocarcinoma | FLT1 | 0.2009 | 0.1530 | 0.5 |
| lung adenocarcinoma | MET | 0.7152 | 0.8083 | 0.065 |
| lung adenocarcinoma | PTK7 | 0.0205 | 0.1637 | 0.742 |
| lung adenocarcinoma | TGFBR1 | 0.0792 | 0.6516 | 0.565 |
| pancreatic ductal adenocarcinoma | ACVR1 | 0.3749 | 0.1368 | 0.02 |
| pancreatic ductal adenocarcinoma | DDR1 | 0.0132 | 0.1089 | 0.765 |
| pancreatic ductal adenocarcinoma | DDR2 | 0.2803 | No data | 0.451 |
| pancreatic ductal adenocarcinoma | EPHA2 | 0.0487 | 0.4007 | 0.608 |
| pancreatic ductal adenocarcinoma | ERBB2 | 0.2873 | 0.3314 | 0.314 |
| pancreatic ductal adenocarcinoma | FLT1 | 0.2912 | 0.0334 | 0.177 |
| pancreatic ductal adenocarcinoma | MET | 0.2823 | 0.1387 | 0.373 |
| pancreatic ductal adenocarcinoma | MST1R | 0.0187 | 0.1538 | 0.667 |
| pancreatic ductal adenocarcinoma | PDGFRA | 0.2878 | 0.0608 | 0.236 |
| pancreatic ductal adenocarcinoma | PDGFRB | 0.2884 | 0.0742 | 0.216 |
| pancreatic ductal adenocarcinoma | ROR2 | 0.0357 | 0.2940 | 0.628 |
| squamous cell lung carcinoma | ACVR1 | 0.2781 | 0.0304 | 0.5 |
| squamous cell lung carcinoma | DDR1 | 0.0048 | 0.0393 | 0.731 |
| squamous cell lung carcinoma | EGFR | 0.6017 | 0.5533 | 0.02 |
| squamous cell lung carcinoma | EPHA2 | 0.4726 | 0.1024 | 0.097 |
| squamous cell lung carcinoma | EPHA4 | 0.0076 | 0.0623 | 0.693 |
| squamous cell lung carcinoma | EPHB2 | 0.0109 | 0.0899 | 0.635 |
| squamous cell lung carcinoma | EPHB3 | 0.0083 | 0.0684 | 0.654 |
| squamous cell lung carcinoma | FGFR2 | 0.5056 | 0.0814 | 0.077 |
| squamous cell lung carcinoma | FLT1 | 0.0761 | 0.0725 | 0.597 |
| squamous cell lung carcinoma | IGF1R | 0.0121 | 0.0996 | 0.616 |
| squamous cell lung carcinoma | PTK7 | 0.0046 | 0.0379 | 0.75 |
| uterine corpus cancer | ERBB3 | 0.0138 | 0.1139 | 0.637 |
| uterine corpus cancer | FGFR2 | 0.3476 | 0.0363 | 0.031 |
| uterine corpus cancer | DDR1 | 0.0046 | 0.0380 | 0.788 |
| uterine corpus cancer | ERBB2 | 0.3734 | 0.4533 | 0 |
| uterine corpus cancer | MET | 0.2126 | 0.0652 | 0.304 |

**Supplementary Table S8. Mapping between DepMap lineage annotations and cancer-type labels used for PRISM data in manuscript.**

| **Disease** | **lineage** | **Primary Disease (Subtype)** |
| --- | --- | --- |
| BRCA | BREAST | Invasive Breast Carcinoma |
| CRC | LARGE INTESTINE | Colorectal Adenocarcinoma |
| GBM | Glioblastoma | Glioblastoma |
| HCC | LIVER | Hepatocellular Carcinoma |
| LUAD | LUNG | Non-Small Cell Lung Cancer (Lung Adenocarcinoma) |
| LUSCC | LUNG | Non-Small Cell Lung Cancer (Lung Squamous Cell Carcinoma) |
| OV | OVARY | Ovarian Germ Cell Tumor, Ovarian Epithelial Tumor, Ovarian Cancer |
| PC | PANCREAS | Pancreatic Neuroendocrine Tumor, Pancreatic Adenocarcinoma |
| RCC | KIDNEY | Renal Cell Carcinoma |
| UCEC | ENDOMETRIUM | Endometrial Carcinoma |
